# Both ends of artificial intelligence impacting privacy: a review of violation and protection

**DOI:** 10.3389/frai.2026.1686454

**Published:** 2026-02-18

**Authors:** Nadav Voloch, Ron S. Hirschprung

**Affiliations:** Department of Industrial Engineering and Management, Ariel University, Ariel, Israel

**Keywords:** analysis, artificial intelligence, graph database, machine learning, privacy, review

## Abstract

The intersection of Artificial Intelligence (AI) and privacy presents both significant challenges and opportunities. As AI systems become increasingly embedded in many aspects of our lives, including healthcare, finance, and social networks, and introduce significant concerns regarding privacy issues – the need for effective privacy-preserving mechanisms also grows. This review systematically analyzes 94 research papers in the field of AI and privacy. To model this complex issue, we categorized privacy in AI through a multi-dimensional approach that includes technological domains' privacy actions, privacy-preserving strategies, and AI-privacy interaction directions. A novel technique based on a Graph Database (Neo4J) which is available to the reader was employed to facilitate visualization of the complex relations between the reviewed objects. Moreover, the Graph, which is actually the review, can be queried and updated with future publications. Key findings indicate that AI can be both a potential threat to privacy, for example due to inference risks and data exploitation, as well as a tool for enhancing privacy through techniques such as federated learning and differential privacy. The study highlights regulatory, ethical, and technical challenges, emphasizing the need for interdisciplinary collaboration.

## 1 Introduction

Privacy is a multifaceted concept spanning personal, social, and technological dimensions (Mulligan et al., 2016). It usually revolves around the right of individuals to control information about themselves and to decide how and to what extent this information is communicated to others (Solove, 2004). In the digital era, privacy challenges stem mainly from the collection, use, and dissemination of personal data by various entities, including governments, corporations, and other individuals. Effective privacy protection measures must navigate the delicate balance between individual rights and technological benefits.

Privacy is perceived as a human right in Western countries; therefore, privacy regulations have been strengthened globally to address the increasing concerns vis-à-vis personal data protection. These regulations aim to provide individuals with greater control over their personal information and impose stricter requirements on organizations regarding data handling practices (Greenleaf, 2019). The most prominent example is the General Data Protection Regulation (GDPR) (Regulation (EU) 2016). The GDPR is a regulation for data protection and privacy, initiated by the European Union (EU), and affects most of the commercial companies, government institutions, and other sectors that process personal information of individuals. This regulation applies by virtue of public international law, thus it has a deep meaningful effect on many aspects that involve the handling of private data.

In general, and specifically for the purpose of this review, it is important to clearly define the term privacy, and its borders. Specifically, we would also like to distinguish between privacy and security, which are often confused. Privacy can be understood as the claim of individuals, groups, or institutions to determine for themselves when, how, and to what extent information about them is communicated to others. Solove (2005) expanded the concept of privacy to include not just individual rights but also the broader societal implications of information control. Privacy in the technological context refers to the ability to control the collection, use, and dissemination of personal data, particularly in digital systems where information can be rapidly transmitted, processed, and stored (Langheinrich, 2001). While privacy and security are closely related, they are not identical. Differentiating between them is essential, for example, in situations where there is a trade-off between the two concepts, or when a regulator imposes rules concerning privacy or security or both. This differentiation can better facilitate the selection of proper means of defence. A methodology for differentiating between privacy and security was introduced based on four components: individual target, nature of the cost, existence of a trade-off, and consent (Hirschprung, 2023). This distinction is important and relevant to this review, in light of the fact that tit focuses on privacy, and not on security.

Protecting private data (and specifically, private users’ data) from breaches and unwanted usage is essential to maintain trust in digital platforms. It requires strong defense measures and well-formed data management. For this reason, privacy has been extensively researched in the last years. For example, exploring the critical issue of privacy in Online Social Networks (OSNs), emphasizes the importance of an integrated approach to effectively manage privacy in OSNs (Voloch et al., 2021). Another example of data privacy problems in the digital age is the way that traditional legal frameworks struggle to keep pace with the rapid evolution of technology, leading to incomplete and sometimes inadequate privacy protection (DeVries, 2003). Researchers have proposed various solutions to address privacy concerns, with focus on enhancing data security and user control over shared information.

One approach involves implementing privacy-preserving algorithms, such as differential privacy, which adds noise to data to protect individual identities while allowing aggregate data analysis (Dwork, 2006). Another solution emphasizes decentralized social network architectures, leveraging blockchain technology to provide secure, transparent, and user-controlled data management (Zyskind et al., 2015).

Since the emergence of social networks and the massive use of personal data in everyday digital uses, the subject of privacy has become more and more important. Baruh et al. (2017) showed that users who are concerned about privacy are less likely to use online services and share information, and more likely to employ privacy-protective measures. These concerns have been rising in recent years, as data theft, data manipulation, and other forms of data breaches increasingly occur. These incidents and recent awareness of users, combined with the vast amount of private data that is available digitally online, have resulted in an increase in privacy concerns. This issue of trust is highly relevant to the combination of privacy and the emerging Artificial Intelligence (AI), since we do not necessarily know the sources and abilities of different AI mechanisms, and how they affect data privacy by their implementations.

AI is now integrated into many sectors, including healthcare, finance, and education, and affects a variety of domains including education, e.g., teacher focused aids that include plagiarism detection for assignments, automated assessments and AI teaching assistance (Holmes and Tuomi, 2022), finance, e.g., neural computing methods that help detect abnormal market behaviors, anomalies or frauds in corporate finance, accounting, insurance and banking businesses (Cao, 2022), and healthcare, e.g., to improve medical diagnosis, to accelerate pharmaceutical revelations and to perform robotic surgery (Shaheen, 2021). AI technology is not just about automating tasks, but also about enhancing decision-making processes with deep data insights. This ability is available due to the fact that AI has the power to interpret tasks from the data provided as input and understand from such input what needs to be done. Moreover, there are new abilities that rely on synthetic data, for example Large Language Models (LLMs) that use synthetic data for training and fine-tuning, in order to achieve more accurate results for user interactions.

AI is a broad field in computer science and other disciplines, aimed at creating systems that can perform tasks that require human intelligence, such as reasoning, learning, and understanding language (Vaswani, 2017). Machine Learning (ML), which may be considered a subset of AI, specifically focuses on algorithms that enable training software based on some data, and improving over time without explicit programming. While AI encompasses a variety of approaches, including rule-based systems that do not learn, ML always involves learning from data to make predictions or decisions. For example, Jakhar and Kaur (2020) discuss the use of AI and ML in dermatology while relying on empirical evaluations. Their research also provides an overview of the use of deep learning (DL) in AI and emphasizes the need for dermatologists to understand these technologies as they become increasingly important in dermatological imaging and diagnosis.

AI has become accessible to the general public mainly through LLMs that imitate human interaction, with the addition of knowledge, abilities, and data resources of a powerful computer. The public release of LLMs rapidly increased their adoption and visibility in the exposure of platforms such as OpenAI’s ChatGPT, Meta’s Llama, and others. These platforms provide the users with the ability to use their natural language and describe tasks and interactions using an interface that is manifested as a friendly chat, to conclude and perform tasks of different complexity levels.

ChatGPT is the leading platform used by the general public, and its abilities and easy-to-use interface has facilitated the extensive surge in its use in LLMs. The rapid integration of AI in everyday life and in numerous fields, as mentioned above, has also introduced challenges and risks, emphasizing the need for responsible AI practices. Issues such as privacy, data governance, transparency, and the safety of AI systems are paramount as we aim to mitigate risks for all stakeholders involved. Therefore, the development and deployment of AI must be handled with care to ensure it benefits society while minimizing or balancing potential harms (Floridi et al., 2018).

The integration of AI into various sectors has raised significant concerns regarding privacy, particularly in terms of data collection, processing, and the potential misuse (Binns, 2018). As AI systems become more sophisticated, the challenge of ensuring that privacy is indeed maintained while harnessing AI’s capabilities becomes increasingly complex (Wachter et al., 2017). Privacy issues in AI involve legal, technological, and ethical aspects. Legally, there are concerns about data protection laws not keeping pace with AI’s capabilities, leading to potential violations of privacy rights as AI systems increasingly use personal data for training and decision-making (Schwartz and Solove, 2014). Technologically, AI models often require large datasets that can inadvertently expose sensitive information, highlighting the need for robust privacy-preserving techniques (Shokri and Shmatikov, 2015). Ethically, there is an ongoing debate about the moral implications of AI’s ability to infer private information from seemingly innocuous data, raising questions about consent and autonomy in digital spaces (Mittelstadt, 2019). AI may be perceived as the ability to slide the curtain that hides private data without breaching or violating any existing law, a source of significant concern.

As the field of AI is constantly changing, consequently, it also introduces new aspects of privacy issues (Carmody et al., 2021). Therefore, It is important to understand how AI and privacy interact in different situations. Again, as mentioned above, when people express concerns about privacy in relation to AI technologies, they are usually referring to security interests rather than interests in privacy (Elliott and Soifer, 2022). Consequently, while these areas are closely related, they should be distinguished from one another, where each introduces unique challenges.

Privacy and AI are inherently interconnected, simply due to the fact that AI systems often rely on vast amounts of personal data to function effectively; and the collection, processing, and storage of this data raise concerns about individuals’ privacy. These concerns particularly deal with how data is used, shared, and protected. AI models, especially in areas such as facial recognition, user profiling, and predictive analytics, can pose risks of unauthorized data access, misuse, or even surveillance. As AI continues to evolve, balancing innovation with privacy protection is crucial to maintain trust and adhere to ethical standards in data handling.

In light of the above, we can argue that privacy and AI collide. Therefore, considering the necessity of privacy as a human right, and the popularity of AI as a highly beneficial tool, their interaction is significant. In this paper, we review the current boundaries and common grounds of research on privacy and AI. We selected research papers in different domains that investigated the relations, effects, and collisions of AI technologies and privacy. The reviewed research are categorized into several classes, and different perspectives, with the aim of perceiving their views on privacy and AI. For the purpose of this review, first we defined the classes, then reviewed 94 papers, and finally created a database that is centric on the complex relations between the classes and the papers.

The objectives of this review are to: (a) Map the domains that are included in Privacy in AI, and the effects of privacy issues in each domain; (b) Define the variety of actions (attack and defense) that are being taken in the domains that involve privacy in AI models and applications; (c) Review and analyze the different approaches, such as Privacy by Design, Privacy shell, etc., practiced by different domains of privacy in different AI models, such as ML, Internet of Things (IoT), Natural Language Processing (NLP), and their applications; and (d) map this field. We used a Graph Database technology (GDB) to present the data in this review to enable a human-friendly presentation, an efficient search based on complex links of this multifaceted data, and also to facilitate future updates by the readers.

## 2 Methodology

### 2.1 General approach

**Figure 1:**
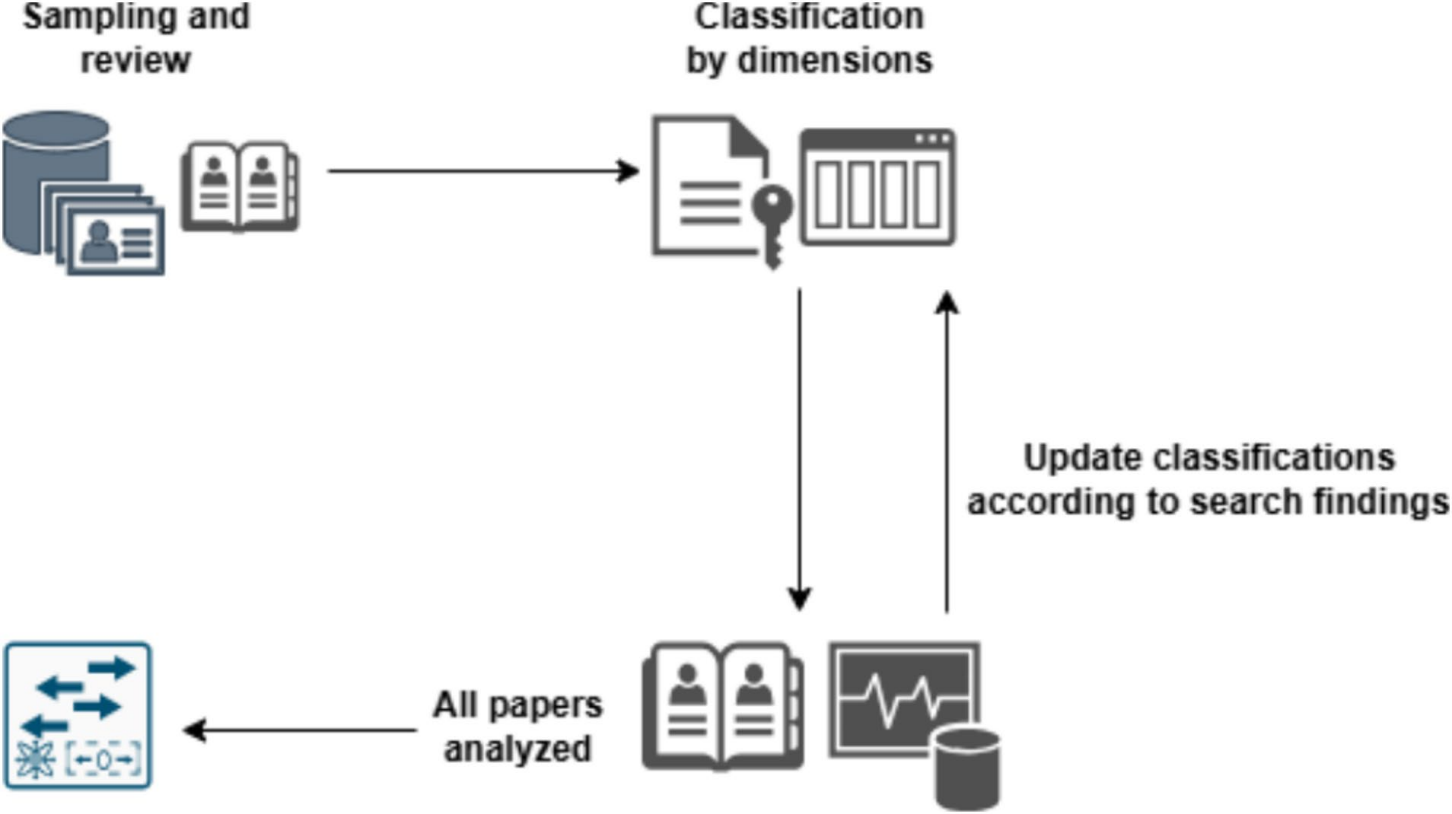
Overview of the methodological process, including sampling, classification, review, and graph-based representation.

In this review, a unique approach was adopted to collect and mainly to organize the findings. The approach is based on the acknowledgement that the data is multi-dimensional by nature. The process is depicted in Figure 1, and includes four major stages as follows:

- Sampling – The first stage included finding several papers that handle the intersections of Privacy and AI. From this sample, general conclusions were drawn regarding the different dimensions, i.e., the different classifications of these subjects, their relation, their approach, etc. This classification was important for the next stages of the process and is dynamic and modular. In actuality, it was changed and updated according to the advancement in the findings in the next stages of the process. The sampling stage was the first stage of the entire process.
- Classification – The second stage included the classification of the different papers into values according to four dimensions, as follows: (i) the technological domain of the paper; (ii) the privacy actions described in the paper; (iii) the privacy approach described in the paper; and (iv) the AI - privacy relation direction described in the paper. Detailed explanations and different values of these domains are described below.
- Thorough search, review and analysis – In this stage, a thorough search of papers was conducted using different platforms such as Google Scholar, IEEE Xplore, ACM Digital Library, SpringerLink, PubMed, and Scopus. The search was performed by specifying relevant keywords that adhere to the different dimensions. The search involved delving deep into the papers and analyzing their content with intake on Privacy and AI according to the different dimensions described in the second phase. These dimensions were also updated dynamically with the progress in the analysis, in a modular way that suited the findings. This stage constituted the core of the review and preceded the graph-based presentation.
- Graph presentation – After completing the review and analysis of the papers, the final stage comprised defining a Graph Database (GDB), where all the papers and the classifications (dimensions) were constructed. Neo4J was selected as the GDB engine. This software provides a unique graphic perspective of the different dimensions and their values, with links to the papers reviewed. The graph may be graphically presented, queried, and updated later as well.

This article is designed as a scoping review combined with an evidence-mapping study, rather than a traditional systematic review. Because the literature on AI and privacy includes conceptual, methodological, and empirical studies with substantial heterogeneity, a scoping approach is most appropriate for identifying the boundaries of the field, mapping key themes, and organizing diverse evidence types. The evidence-mapping component is operationalized through the four-dimensional classification (Domain, Action, Approach, Direction) and the accompanying Neo4J graph database, which visually and analytically represents relationships across heterogeneous studies. Accordingly, the goal of this review is to map, categorize, and synthesize the landscape, rather than to evaluate effect sizes or empirical outcomes, which would be required in a formal systematic review.

The body of literature on AI and privacy is inherently heterogeneous and includes both conceptual/theoretical contributions and empirical or experimental studies. Because the purpose of this work is to map the breadth of research at the intersection of AI and privacy, rather than to synthesize effect sizes or evaluate interventions, both evidence types were intentionally included. Conceptual studies contribute essential definitions, frameworks, and normative analyses that shape privacy interpretations in AI contexts, while empirical studies provide concrete evaluations of privacy attacks, defenses, user behavior, or system performance. The four-dimensional classification model was explicitly designed to accommodate this diversity without forcing the literature into a single methodological mold.

Because the literature on AI and privacy includes both conceptual analyses and empirical evaluations, the review intentionally includes both types of studies. Conceptual studies inform definitions and theoretical frameworks, while empirical studies provide concrete evidence of attacks, defenses, and technical behavior.

### 2.2 Searching for the papers

Building on the general methodological framework outlined above, the next step involved implementing the structured search process across the selected academic databases.

To identify relevant research papers, a deep search strategy was designed, with the aim of locating studies that explored the intersection of privacy and AI, with particular emphasis on the technological domains identified in the classification framework presented in the next subsection. The searches were conducted across several prominent academic databases, including Google Scholar, IEEE Xplore, ACM Digital Library, SpringerLink, PubMed, and Scopus, to ensure comprehensive coverage of the literature. The search terms were carefully crafted to reflect the specific privacy challenges, solutions, and methodologies associated with different AI applications. For instance, terms such as “privacy in Large Language Models” and “GPT privacy” were used for LLMs. Similarly, queries were constructed for other domains, such as “privacy in computer vision” and “AI privacy in IoT devices.” The purpose was to capture the nuances of these intersections, as in queries like “NLP and privacy challenges” or “Online Social Networks and privacy vulnerabilities.” This allowed us to map the vast landscape of research on privacy in AI across domains like computer vision, speech recognition, IoT, online social networks (OSNs), and databases.

The search strategy evolved iteratively as insights emerged during screening into the dimensions and values that aligned with the classification framework. Papers that explicitly addressed privacy issues within these technological domains were included, while those focused solely on AI advancements without privacy considerations or on general privacy issues unrelated to AI were excluded. After performing the searches, a thorough manual review of the titles, abstracts, and keywords was conducted to ensure each paper’s relevance to the research objectives and dimensions. This meticulous process enabled us to build a robust and representative dataset of scientific papers that served as the foundation for the analysis. By iteratively refining the search terms and reviewing the results, the approach ensured that the review remains comprehensive and aligned with the goals of capturing the diverse intersections of privacy and AI across multiple domains.

#### 2.2.1 Guidelines

To classify the relevant papers, a number of research questions were defined as follows:

1. What are the different domains of AI that have privacy issues or that involve privacy?
2. What are the common privacy problems in every domain of AI?
3. What are the specific privacy violations that happen in different AI domains, models and applications?
4. What privacy protection strategies and techniques are used in different domains in AI models and applications?

There could be several values for these research questions, as well as several values for the four different dimensions described below, for each paper reviewed.

#### 2.2.2 Search parameters and screening details

To increase the methodological transparency, additional parameters of the search process are provided. The literature search encompassed publications from 2000 to 2025, a period chosen to reflect the emergence of modern machine learning, deep learning, and large-scale AI systems that are central to contemporary privacy challenges. Only papers written in *English* were included, as English dominates the scholarly output in AI and privacy research. Screening was conducted in two steps: an initial automated filtering by keywords and relevance, followed by a manual review of the full text. While the review was divided between the authors, due to the exploration and mapping-oriented nature of this study, all coding decisions and classifications were revisited iteratively by all authors to ensure internal consistency. The review was finalized on May 15th, 2025, which served as the cutoff date for all database queries. All searches were executed using explicit Boolean expressions and domain-specific query variants (e.g., for LLMs, IoT, OSN, computer vision), ensuring consistency across Google Scholar, IEEE Xplore, ACM Digital Library, SpringerLink, PubMed, and Scopus. This timestamped cutoff ensures that the review reflects the complete body of literature available up to that date and enables full reproducibility of the search strategy.

The corpus intentionally includes both conceptual studies (e.g., theoretical analyses, frameworks, regulatory interpretations, and privacy-by-design methodologies) and empirical studies (e.g., experiments, model evaluations, attack demonstrations, and performance assessments of privacy-preserving techniques). This mixed-evidence inclusion is consistent with the goals of a scoping review and evidence-mapping study, which aim to synthesize heterogeneous forms of knowledge rather than restrict the analysis to one methodological tradition. The four-dimensional classification model was designed precisely to accommodate and systematically organize such diverse study types. All included papers, whether conceptual or empirical, were required to provide clear relevance to AI-privacy interactions and sufficient detail to support classification along the four dimensions.

To further clarify the search process, the exact search syntax, time range, screening counts, and structured inclusion/exclusion criteria are specified below:

Search queries were tailored for each database, but they were based on a consistent Boolean structure that combined AI domain terms with privacy-related concepts. The core template was:

(“Artificial Intelligence” OR “Machine Learning” OR “Deep Learning” OR “Large Language Model” OR NLP OR “Computer Vision” OR “Speech Recognition” OR IoT OR “Online Social Networks”)
AND
(privacy OR “data protection” OR “privacy-preserving” OR “privacy attack” OR “privacy violation” OR “privacy by design” OR “privacy shell” OR “PPDM”).

The following are examples of domain-specific queries:

- LLMs: (*“Large Language Model” OR “GPT” OR “ChatGPT”) AND (privacy OR “data leakage”*)
- Computer Vision: (*“computer vision” AND (privacy OR “re-identification” OR “face recognition privacy”*))
- IoT: (*IoT AND (privacy OR “edge privacy”*))
- OSN: (*“online social networks” AND (privacy OR “profiling” OR “user data misuse”*))

These queries were applied across Google Scholar, IEEE Xplore, ACM DL, SpringerLink, PubMed, and Scopus.

The inclusion criteria required that a study will: (1) explicitly address the intersection of artificial intelligence and privacy; (2) involve at least one technological domain from the four-dimensional framework (e.g., machine learning, large language models, natural language processing, computer vision, speech recognition, IoT, online social networks, or privacy-preserving databases); (3) describe concrete privacy actions such as attacks, defenses, vulnerabilities, or regulatory mechanisms; (4) provide sufficient methodological or conceptual detail to support coding into the Domain-Action-Approach-Direction model; and (5) be an English-language academic publication (peer-reviewed article, conference paper, or reputable preprint).

Studies were excluded if they met one or more of the followings: (1) examined AI technologies without a substantive privacy component; (2) discussed privacy issues unrelated to AI; (3) consisted solely of ethical or legal commentary without technical content; (4) were non-academic sources such as blogs, news items, or policy briefs; (5) lacked methodological detail required for reliable classification; and (6) were written in languages other than English.

To ensure that all included papers directly addressed the intersection of Artificial Intelligence (AI) and privacy, we implemented a structured relevance-screening protocol in addition to the standard inclusion/exclusion criteria. Each paper was required to (a) involve at least one AI technological domain (e.g., ML, LLMs, NLP, computer vision, IoT, OSNs), and (b) articulate a concrete privacy component, such as a privacy violation, defense mechanism, regulatory implication, or privacy-preserving strategy. Papers that referenced AI only superficially or discussed privacy in a purely abstract or legal manner without connection to AI technologies, were removed during title/abstract screening or full-text review. This step ensured that all retained studies addressed the AI-privacy intersection in a substantive and technically meaningful way.

Although the review does not apply a formal quantitative quality-assessment tool due to the conceptual and methodological heterogeneity of the literature, we incorporated a basic qualitative appraisal to enhance internal validity. During full-text screening, each paper was evaluated on: (1) clarity of its research question or objective; (2) transparency of its data, methods, or conceptual framework; (3) explicitness of the privacy construct examined (e.g., violation type, defense mechanism, risk model); (4) adequacy of the AI component (e.g., real models, inferred models, validated algorithms, or conceptual analysis grounded in AI methods); and (5) methodological coherence relative to the paper’s goals. Papers that failed to meet these minimum criteria were excluded. These quality-assurance steps, combined with cross-checking by both authors, strengthened the internal validity of the final corpus and ensured that the resulting synthesis accurately reflects the genuine intersections between AI technologies and privacy concerns.

### 2.3 Classifications

After identifying the complete set of eligible studies, we organized them using a structured four-dimensional classification system to enable systematic comparison and evidence mapping.

As mentioned above, in this research the boundaries and common grounds of AI and privacy were addressed in a unique perspective considering four different dimensions. These dimensions that define the taxonomy of the papers, and their values are as follows:

#### 2.3.1 Domain

The technological domain of a research paper refers to the specific field or area of technology that the study addresses, encompassing the relevant methods, tools, applications, and innovations central to the research focus.

It defines the context within which the technological contributions or advancements are analyzed or developed. The values that were identified for this dimension are: LLM’s, ML, NLP, computer vision, speech recognition, IoT, OSNs, and databases (DB’s).

#### 2.3.2 Actions

The privacy actions described in the paper refer to the specific measures, techniques, or strategies proposed or analyzed to safeguard data privacy, mitigate privacy risks, or address privacy-related challenges in the context of cybersecurity. These actions encompass the practical implementations or theoretical approaches outlined to enhance privacy protection.

The values that were identified for this dimension include: attacks, defense, awareness, vulnerabilities, threats, and regulations.

#### 2.3.3 Approach

The privacy approach described in the paper refers to the overarching methodology, framework, or conceptual strategy adopted to address privacy concerns, analyze privacy issues, or develop privacy-enhancing solutions in the context of cybersecurity. This approach outlines the guiding principles or theoretical foundation underlying the research. The values that were identified for this dimension are: Privacy by Design (PbD), privacy shell, hybrid (PbD + Shell), advisory, and Privacy Preserving Data Mining (PPDM).

#### 2.3.4 Direction

The AI-privacy relation direction described in the paper refers to the specific way the study explores, analyzes, or addresses the intersection between AI and privacy, including the implications, challenges, or opportunities that arise from their interaction. This direction highlights the focus of the research in connecting AI advancements with privacy considerations.

The values that were identified for this dimension are: harnessing AI to protect privacy, AI as a threat to privacy, AI usage that includes privacy, and applying privacy to AI.

**Figure 2:**
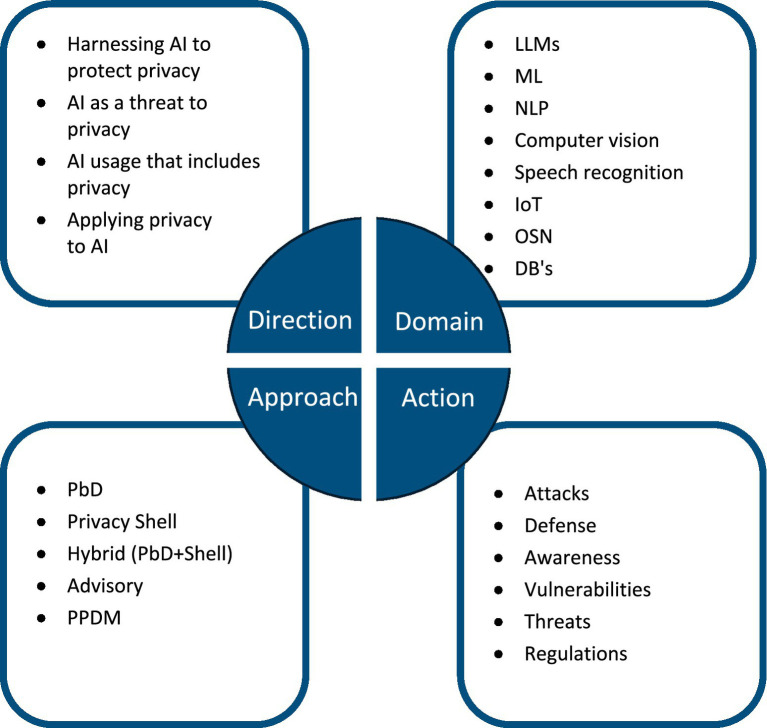
The four-dimensional classification framework that was applied in the review (domain, action, approach, direction) and its respective values. The space of values for each dimension (domain, actions, approach, and direction) is briefly presented herein.

All the dimensions and their values are illustrated in Figure 2.

#### 2.3.5 Domain

LLMs - are advanced AI systems trained on extensive text datasets to understand and generate human-like text. They are widely used for tasks like content creation, language translation, and coding assistance. For example, OpenAI’s GPT-4 can answer complex questions, generate essays, or even assist in debugging code, showcasing its versatility in various domains.

ML - enables computers to learn patterns and make decisions and predictions based on data. It is the backbone of many AI applications, from recommendation systems to fraud detection. For instance, an ML algorithm can analyze customer purchase histories to suggest personalized product recommendations on e-commerce platforms.

NLP - focuses on the interaction between computers and human language, facilitating tasks such as translation, text summarization, and sentiment analysis. A notable example is a customer feedback analysis system that identifies whether reviews are positive or negative, that help businesses better understand customer satisfaction.

Computer vision - enables machines to interpret and process visual information that usually is captured by cameras. This technology is used by applications like facial recognition, autonomous vehicles, and medical imaging. For example, a self-driving car uses computer vision to detect and classify objects on the road, such as pedestrians, vehicles, and traffic signs.

Speech recognition - converts spoken language into written text, enabling technologies like voice assistants, transcription services, and voice-controlled devices. A common example is using virtual assistants like Siri or Alexa to perform tasks such as setting reminders or playing music based on voice commands.

IoT - refers to the interconnected network of devices that communicate and exchange data. These devices include smart home systems, wearable health trackers, and industrial sensors. For instance, a smart thermostat can learn user preferences and automatically adjust home temperatures to optimize comfort and energy efficiency.

OSNs - are platforms where people interact, share content, and build communities. They are used for personal connections, brand promotion, and data analysis. For example, businesses analyze trends and customer opinions on Twitter to refine their marketing strategies and engage with their audience.

Databases - store and manage data systematically, supporting applications like banking systems, e-commerce platforms, and inventory management. For example, an online store uses a database to store product details, customer information, and transaction records, to ensure seamless operations and personalized user experiences.

#### 2.3.6 Action

Attacks *-* are deliberate actions aimed at compromising individuals’ or organizations’ private information. These attacks exploit weaknesses in systems or human behavior. For example, a phishing attack tricks users into revealing sensitive information, such as login credentials or credit card details, by masquerading as a legitimate entity.

Defenses - are measures to protect data and prevent unauthorized access to information. These include encryption, access controls, and regular security updates. For instance, end-to-end encryption in messaging apps like WhatsApp ensures that only the sender and recipient can read the messages, safeguarding privacy from potential eavesdroppers.

Awareness - involves educating individuals and organizations about the importance of protecting sensitive data and recognizing potential threats. For example, campaigns during Cybersecurity Awareness Month teach users to create strong passwords, avoid suspicious links, and recognize phishing attempts to reduce privacy risks.

Vulnerabilities - are weaknesses in systems, applications, or human practices that can be exploited to breach privacy. For example, outdated software without security patches may have flaws that hackers can exploit to gain unauthorized access to personal or organizational data.

Threats - are potential risks to the confidentiality, integrity, or availability of data. These can derive from cybercriminals, insider threats, or even poorly secured devices. For instance, the use of unsecured public Wi-Fi can constitute a threat, because attackers can intercept sensitive data transmitted over the network.

Regulations - are laws and guidelines which are mandatory, designed to protect personal data and ensure compliance with ethical practices. Examples include the General Data Protection Regulation (GDPR) in the European Union, which mandates strict control on how organizations handle user data and empower individuals with the right to access and delete data.

#### 2.3.7 Approach

Privacy by Design (PbD) - is an approach that incorporates privacy protection directly in the development of systems and processes and is applied, beginning with the design stage. It focuses on proactive measures, minimizing data collection, and ensuring secure handling throughout the data lifecycle. For example, a fitness tracking app built with PbD principles might collect only necessary data, such as step counts and calories, while avoiding sensitive information such as precise GPS locations unless explicit consent is given by the user.

Privacy shell - is a conceptual framework or layer that enhances privacy protection by acting as an intermediary between the user and the system. It employs measures like anonymization, access control, and policy enforcement. For instance, an online survey platform may use a privacy shell to anonymize survey responses before storing them, to ensure that even if the data is leaked, individual participants cannot be identified.

Hybrid approach - combines the principles of PbD with the added protection of a privacy shell to create a robust privacy strategy. This ensures that privacy is integrated into the system from the beginning while dynamically applying additional layers of security. For example, a healthcare system using a hybrid approach might limit data collection to essential patient information (PbD) while encrypting the data and allowing access only to authorized personnel by means of a privacy shell.

Advisory approach - involves providing guidance to users and organizations on the best privacy practices, compliance requirements, and policy implementation. This approach is aimed at improving awareness of and adherence to privacy standards. For instance, a corporate advisory service might audit a company’s data handling practices and suggest updates to comply with regulations such as GDPR, e.g., by implementing access logs or revising privacy policies.

Privacy-preserving data mining - focuses on extracting meaningful insights from data while safeguarding individual privacy. Techniques like anonymization, differential privacy, and secure computation are commonly used. For example, a medical dataset that includes sensitive information about patients, which is released for research purposes – may be anonymized by using the *k*-anonymity technique.

#### 2.3.8 Direction

Harnessing AI to protect privacy - AI can be a powerful tool for enhancing privacy by automating data protection and enforcing compliance with privacy regulations. For example, AI-driven algorithms can detect potential data breaches in real time by analyzing unusual activity patterns, alerting administrators, and taking immediate action to secure sensitive information. Additionally, AI-powered tools can anonymize data before sharing it for research or business analysis, to ensure compliance with regulations like GDPR.

AI as a threat to privacy *-* While AI offers many benefits, it also poses significant risks to privacy. AI systems can collect and process massive amounts of personal data, often without explicit user consent. For instance, facial recognition technology used in public spaces can track individuals’ movements and identities, which raises concerns about surveillance and the potential misuse of personal data. Similarly, AI algorithms used for targeted advertising can infer sensitive information about users based on their online behavior, sometimes exposing more about individuals than they had intended to share.

AI usage that includes privacy *-* AI systems can be designed to incorporate privacy-preserving techniques, ensuring that their usage aligns with ethical standards. For example, an AI chatbot used by a healthcare provider could be programmed to encrypt conversations and delete sensitive information after a session ends, thereby protecting patient confidentiality. These measures allow AI to operate effectively while maintaining user trust and compliance with privacy laws.

Applying privacy to AI - Applying privacy principles to AI involves designing and implementing systems that prioritize data security and minimize risks to user information. This can include measures such as differential privacy, which ensure that data analysis results do not reveal individual user’s information. For instance, an AI model trained on users’ data to improve recommendation system can apply differential privacy to ensure that no single user’s data can be reverse-engineered or exposed, even in the case of data breach.

### 2.4 Justification and development of the four-dimensional classification model

The four-dimensional model (Domain-Action-Approach-Direction) was developed to capture the multidimensional nature of privacy-AI interactions. These dimensions were selected after an exploratory sampling stage, during which patterns repeatedly emerged across heterogeneous studies. Conceptually, each dimension represents a distinct layer of analysis:

- Domain captures the technological context in which privacy issues arise,
- Action captures the type of privacy-related activity (e.g., attack, defense, regulation),
- Approach describes the conceptual framework used to address privacy, and
- Direction reflects how the study positions AI in relation to privacy (e.g., beneficiary, threat, neutral mechanism).

Together, these dimensions offer a structured map for comparing fundamentally different study types without forcing them into a single evaluative frame. Their separation is therefore intentional: privacy problems, privacy strategies, conceptual philosophies, and AI-privacy relational dynamics operate at different analytical levels and collapsing them would obscure rather than clarify the landscape.

### 2.5 Interrelations among dimensions

Although analytically distinct, the dimensions interact frequently in the literature. For example, studies in the ML or LLM Domains often combine attacks (Action) with privacy-preserving data mining (Approach) and frame AI as both a threat and a tool for defense (Direction). IoT studies often connect vulnerabilities with regulations and privacy-by-design approaches. The graph database highlights these cross-dimensional overlaps, making visible clusters of papers where certain dimension pairs co-occur. This interwoven pattern further justifies a multi-dimensional framework rather than a single-axis taxonomy.

### 2.6 Coding protocol and consistency assurance

To ensure methodological rigor, we implemented a structured coding procedure. After the initial exploration phase, both authors independently coded a subset of papers using the four-dimensional schema. Disagreements were discussed and resolved, leading to refinement of the definitions for each dimension and clearer operational criteria. These refined definitions were then applied consistently to the full dataset. Although formal inter-rater reliability statistics were not calculated due to the conceptual heterogeneity of the publications, all coding decisions were cross-checked by both authors to maintain consistency and reduce subjective bias. The publicly accessible Neo4J graph additionally provides transparency, allowing external readers to inspect how each paper was classified and identify potential inconsistencies.

### 2.7 Graph presentation

A multi-dimensional model well describes data that is classified by more than one factor. Nonetheless, it introduces the challenge of comprehending all of the factors. Since the sole purpose of a review paper is to provide information to individuals, and given that this information might be accessed from different angles (in practical terms the dimensions) – the above-mentioned challenge is significant. To address this challenge, a mathematical graph presentation approach was adopted. A mathematical graph is a structure comprising a set of vertices (also known as nodes or points) and a set of edges (also called links or lines) that connect pairs of vertices (Hamilton et al., 2017). These edges can be undirected, indicating a bidirectional relationship, or directed, signifying a one-way connection. For example, a graph that represents the friendship relations between Facebook friends is undirected because if A is a friend of B then B is necessarily a friend of A and vice versa, and the direction is insignificant. On the other hand, a graph that represents a road map is directional because if traffic is enabled from A to B, traffic is not necessarily enabled from B to A, and thus the direction is significant. This framework allows graphs to model various systems where entities are interconnected, such as social networks, computer networks, or biological networks. While relational databases (RDBs) are better for accumulated data, the graph structure enables a convenient and efficient search for complex relations; and thus, best suits the purpose of representing papers by their different dimensions.

The Neo4J database engine was selected to implement the graph database (Neo4J, 2024). Neo4J is a graph database management system (GDBMS), designed to efficiently store, query, and analyze relationships between data. Unlike traditional RDBs, Neo4j represents data as a graph, i.e., nodes and relations as described above. Neo4J is a directional GDBMS, i.e., each relation has a direction as explained above. A direction is not always necessary, and while Neo4J necessitates defining a direction for each relation when querying the DB, the direction can be ignored. In the current research, because the relation was defined as *BELONGS_TO* (Neo4J represents data in a natural language grammar-like rules), the direction is defined. Neo4J is a labelled property GDBMS, i.e., each node may have a label, and each relation must have one. The label identifies the type of the node or the relation. In addition, each node or relation may have properties, For example, to describe movies and actors’ relations, nodes may have the labels: *actor* and *movie*; an actor node may have the properties: *name*, *gender*, and *year of birth*; while a movie node may have the properties: *movie*_*name*, and *year*; relations may have the labels: *act_in* (between an *actor* and a *movie* nodes) and *familiar_with* (between two actors) - a *familiar_with* relation may include the property *since*.

In the current research graph, there are four types of nodes, one type for a paper node, and one type for each dimension. A relation between a paper node and a dimension signifies that the paper belongs to this dimension (classification). Each paper node has the properties of title, authors, year, publication platform, and DOI/URL. Table 1 describes the different nodes, their possible connections, and their presentation.

**Table 1:** Neo4J graph representation nodes.

Node type	Contents	May connect to	Size	Color
Paper	Paper name	Domain, action, approach, direction	Big	Blue
Domain	Domain value	Paper, hyperlink to detail domain review	Small	Yellow
Action	Action value	Paper	Small	Red
Approach	Approach value	Paper	Small	Brown
Direction	Direction value	Paper		Green

**Figure 3:**
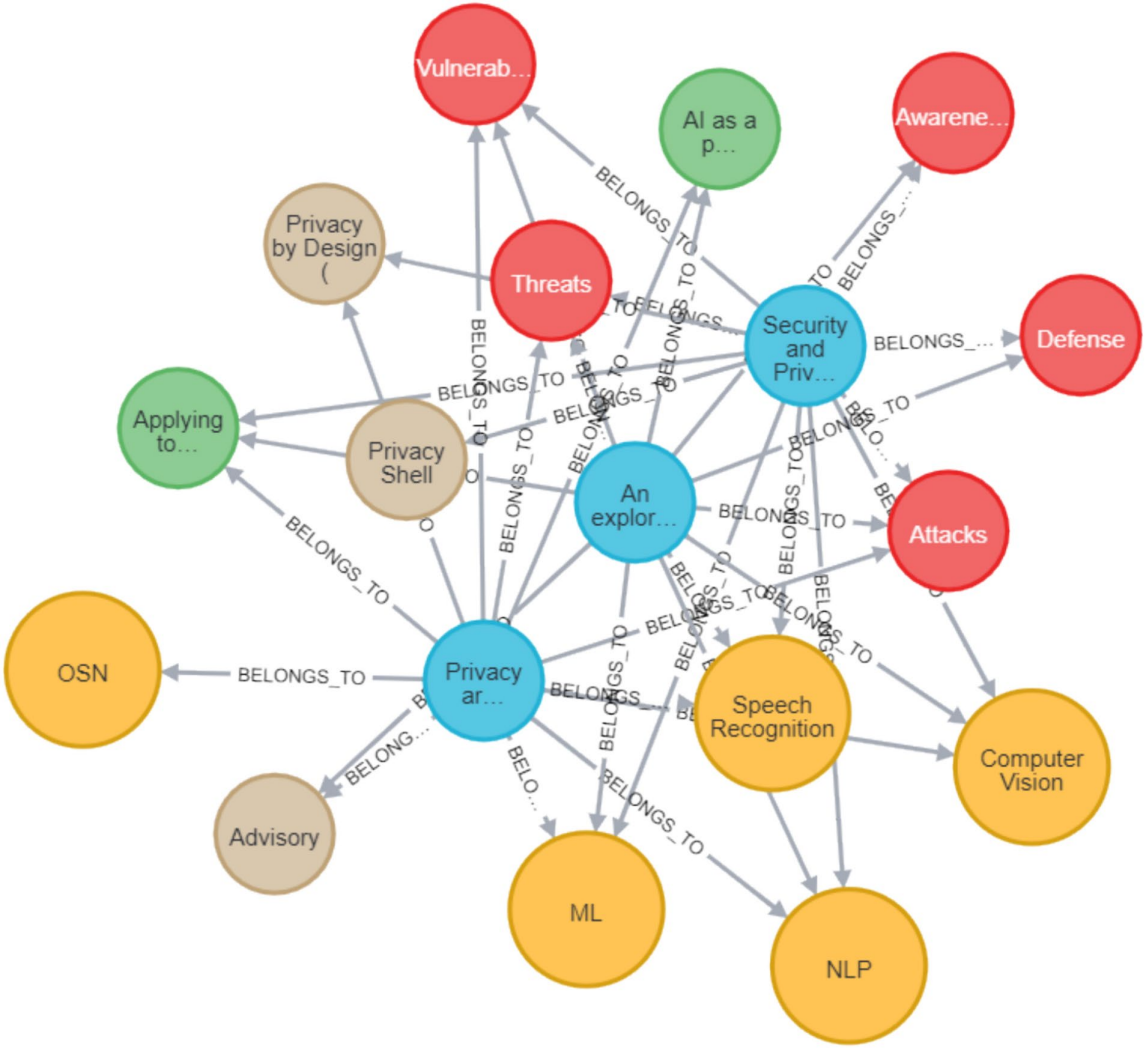
Neo4J graph example, showing three papers and their connections to domain, action, approach, and direction nodes.

First, the raw data which includes the papers’ details, the classifications, and the links between them was uploaded to CSV files. Then, the CSV files were uploaded to the Neo4J environment, to form the graph database. All of the CSV files together with the necessary operating commands in Neo4J Cypher language that were used to create the graph are available in (Voloch, 2024) and indexed with DOI: https://doi.org/10.5281/zenodo.17584342 to maintain persistency, to enable the reader to query as well as update the DB. A simplified and partial example of this sort of graph visualization is depicted in Figure 3. In this example, there are three papers with the connections to their specific dimension values. The blue circles are papers, the red are the different actions, the yellow are the technological domains, the green are the Privacy-AI directions, and the brown are the different approaches. The example shows that OSN (yellow) is only in one paper, but all the other domains are in all three papers. As for the red ones (actions), awareness is only on one paper, defense is in two of them, and all the rest are in all three papers. Figure 3 provides a simplified example of the Neo4J graph structure used in this review. It illustrates how papers are connected to their assigned Domain, Action, Approach, and Direction values, helping the reader understand the structure of the larger evidence map.

### 2.8 PRISMA report

**Figure 4:**
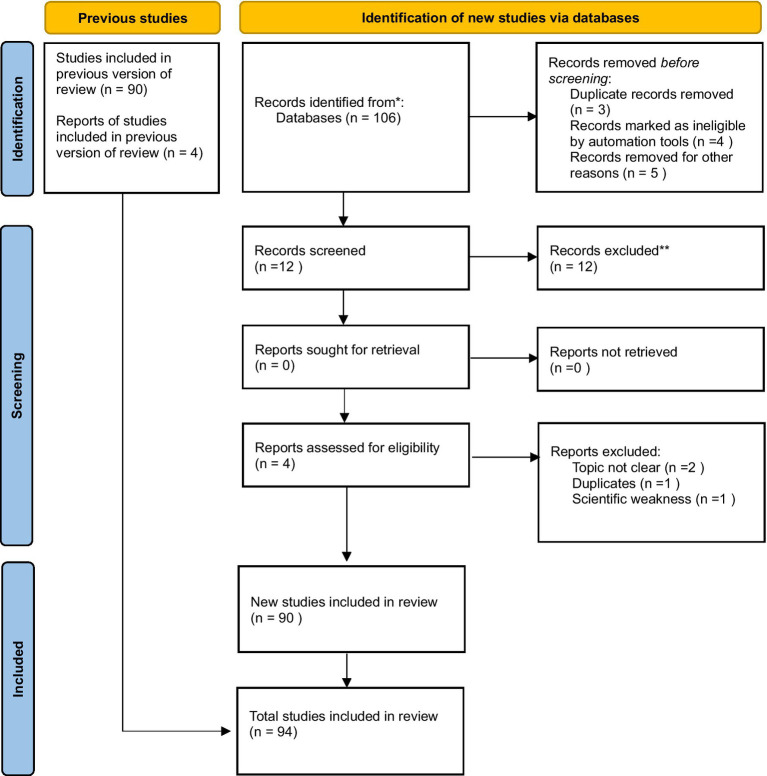
PRISMA 2020 flow diagram for study identification and selection. Source: Page M. J., et al. BMJ, 2021; 372: n71. doi: https://10.1136/bmj.n71. This work is licensed under CC BY 4.0. To view a copy of this license, visit https://creativecommons.org/licenses/by/4.0/.

The review process was structured in accordance with the PRISMA 2020 guidelines for transparent reporting of systematic reviews. The overall workflow, including identification, screening, eligibility assessment, and inclusion, is summarized in a PRISMA flow diagram. Figure 4 summarizes the identification, screening, and inclusion process used in the study. It visually aligns the narrative description with the PRISMA 2020 framework, clarifying how the final corpus of 94 studies was determined.

In the identification phase, records were retrieved from multiple scholarly databases (Google Scholar, IEEE Xplore, ACM Digital Library, SpringerLink, PubMed, and Scopus) using combinations of AI and privacy-related keywords tailored to the technological domains defined in our classification framework (e.g., LLMs, ML, NLP, computer vision, speech recognition, IoT, OSN, and DBs). After removing duplicates, titles and abstracts were screened to exclude papers that did not explicitly address the intersection of AI and privacy (e.g., works focused solely on AI performance or on general privacy without an AI component, non-academic sources, and purely legal or ethical discussions without any connection to AI technologies).

The PRISMA diagram (Figure 4) reflects the supplementary database search conducted after constructing the main corpus of 94 papers from the Neo4J-based classification framework. This supplementary search yielded 106 records, from which 3 duplicates, 4 automation-flagged items, and 5 non-relevant records were removed before screening, leaving 94 records.

From these, 12 records met the initial relevance threshold for title/abstract screening. All 12 were excluded at this stage for reasons documented in the diagram (topic not aligned, duplicates, or methodological limitations).

In parallel, four reports underwent full-text assessment but did not meet the eligibility criteria for inclusion. Consequently, no additional papers were added from this supplementary search, and the final dataset remained 94 papers, which had been established through the main literature mapping and classification workflow.

As mentioned above, full-text assessment was conducted for the remaining records. Studies were included if they: (a) involved at least one AI-related technological domain as defined in Section 2.3; (b) discussed concrete privacy actions, approaches, or AI-privacy relations; and (c) provided sufficient methodological and contextual detail to support classification along the four dimensions (Domain, Action, Approach, Direction). Papers were excluded at this stage if privacy was mentioned only tangentially, if AI was not substantively involved, or if methodological information was insufficient for reliable coding. Reasons for exclusion were documented for each full-text article. The final corpus comprised 94 papers.

**Figure 5:**
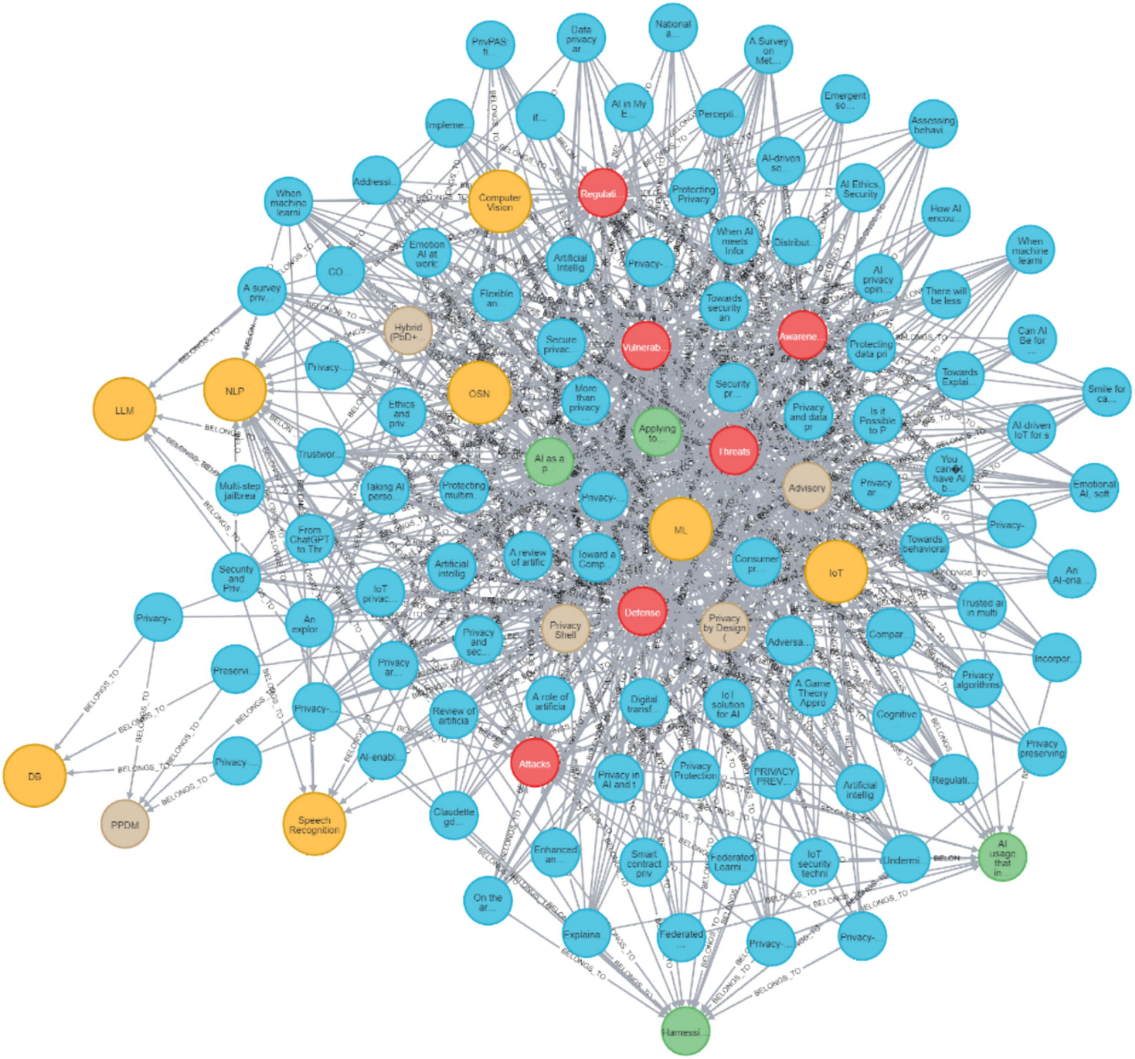
Neo4J visualization of all reviewed papers and their connections to all the different dimensions and values.

**Figure 6:**
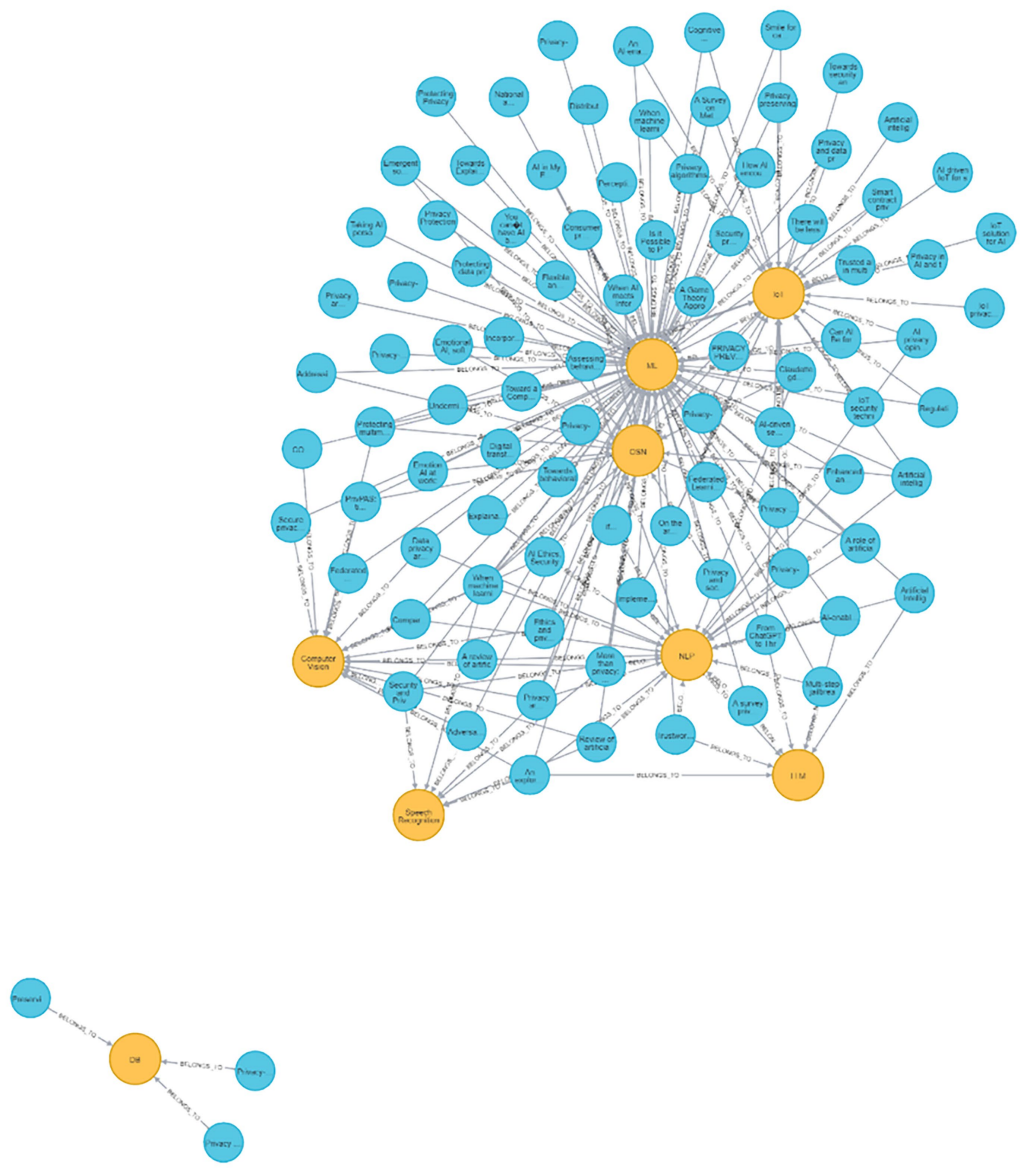
Neo4J visualization of all reviewed papers and their connections to the domain values.

**Figure 7:**
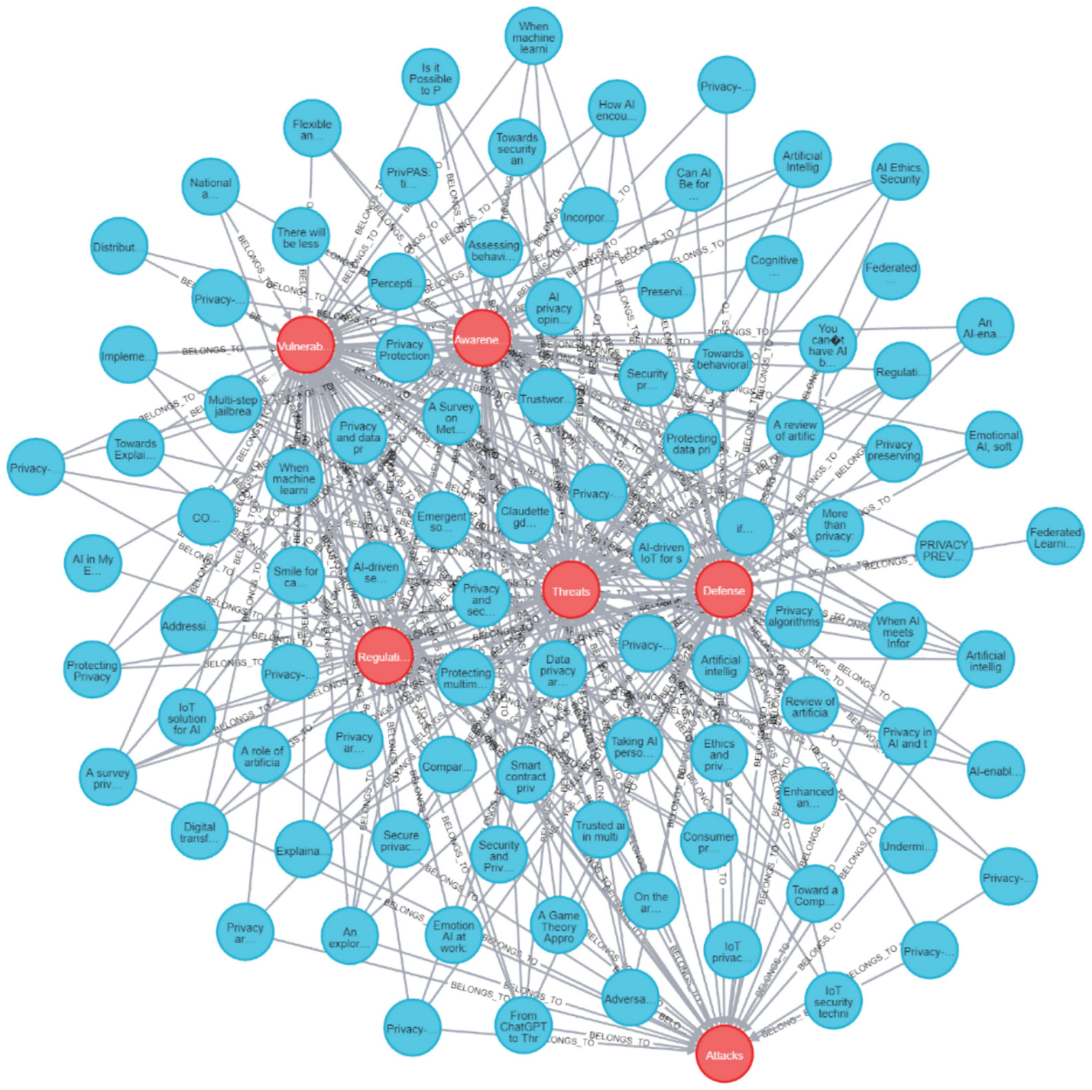
Neo4J visualization of all reviewed papers and their connections to the action values.

**Figure 8:**
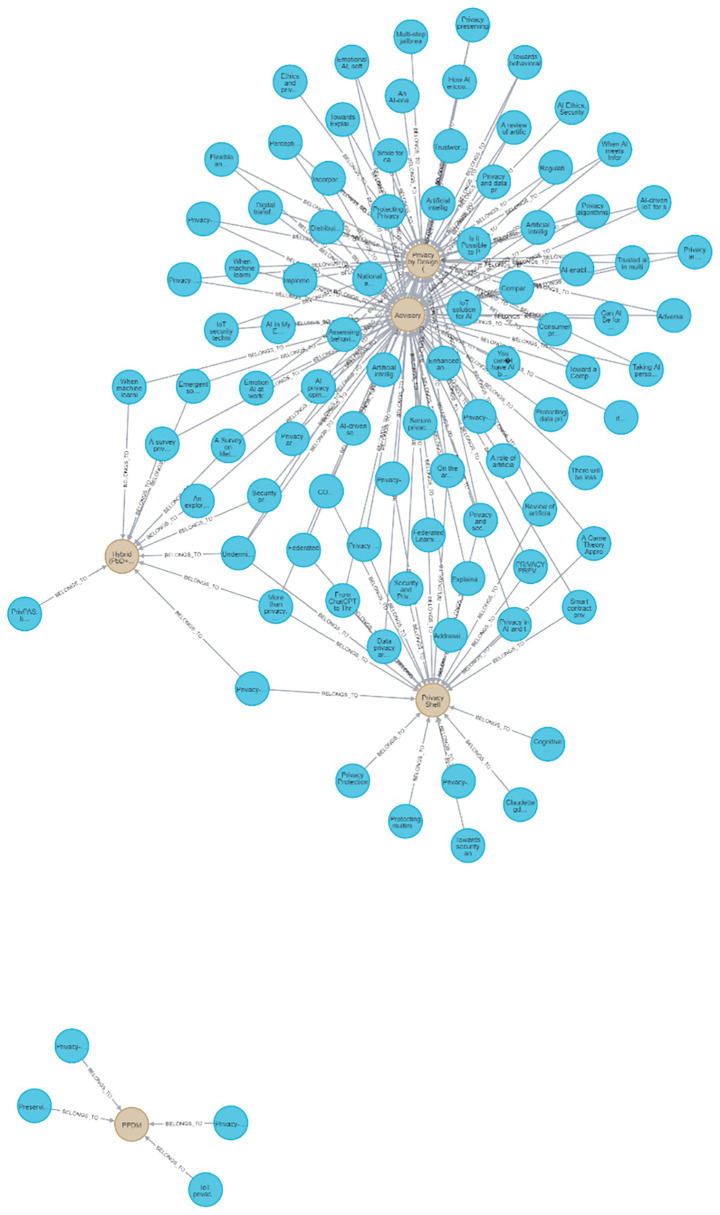
Neo4J visualization of all reviewed papers and their connections to the approach values.

**Figure 9:**
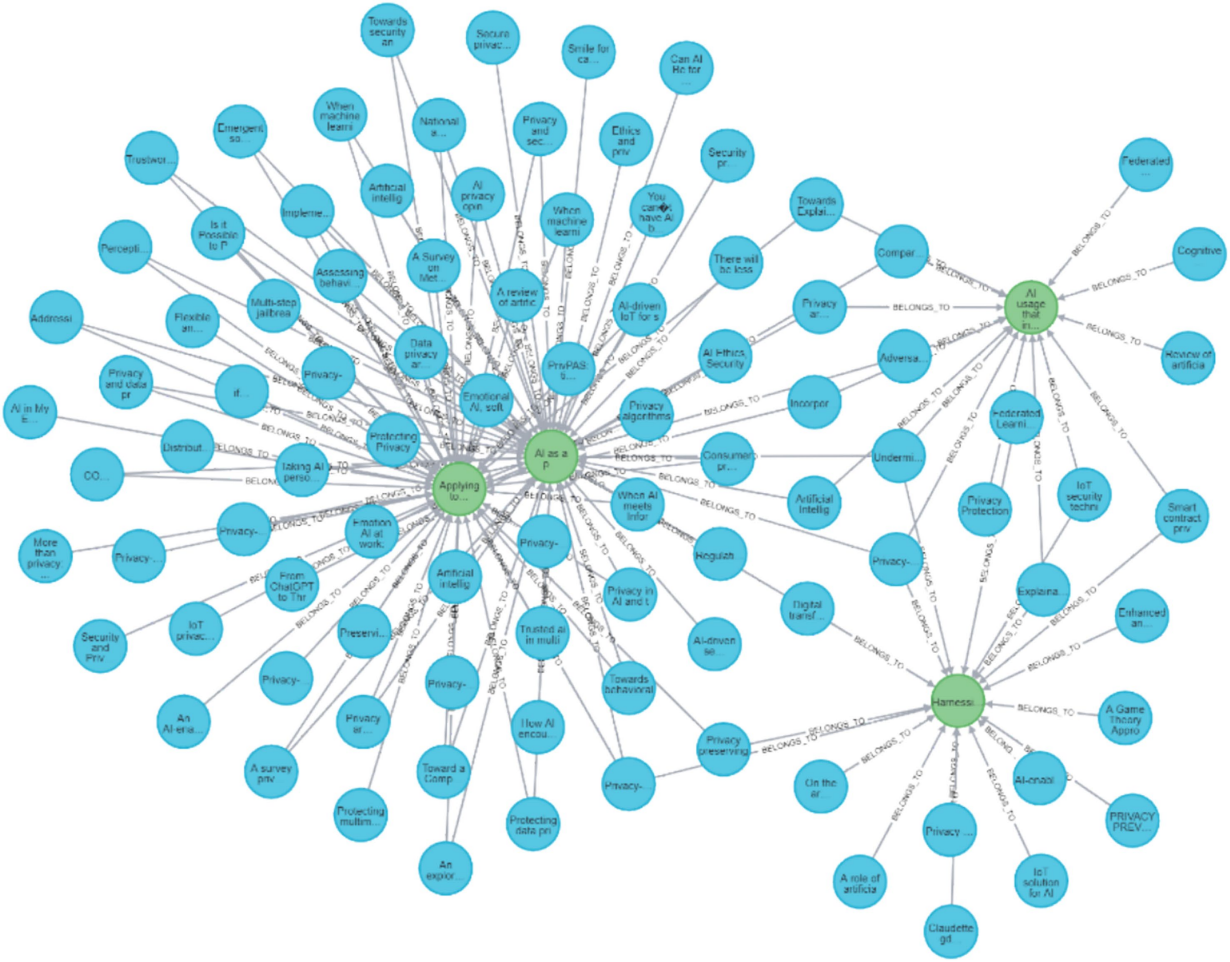
Neo4J visualization of all reviewed papers and their connections to the direction values.

**Figure 10:**
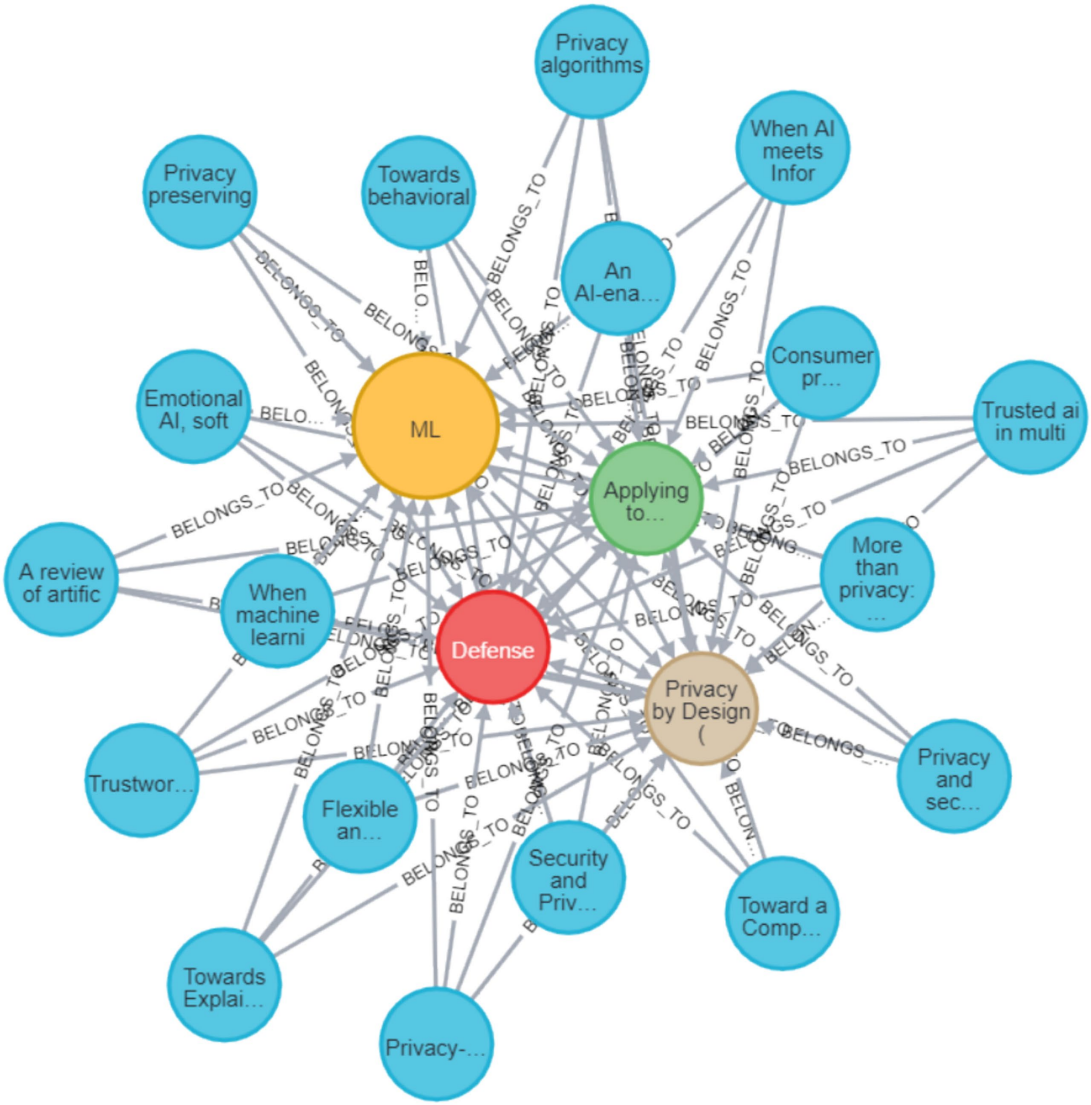
A graphic representation produced by querying the GDB for all the papers that are in the *ML* domain, that include the action of *defense*, follow the PbD approach, and their AI-privacy direction is a*pplying privacy to AI.*

In line with the mapping and taxonomy-building objectives of this work, no quantitative meta-analysis was conducted. Instead, we applied a structured, qualitative appraisal of each included study, focusing on clarity of research questions, transparency of data sources, appropriateness of AI methods, explicitness of privacy constructs (e.g., types of violations, defenses, or regulatory context), and limitations acknowledged by the authors. These elements were captured in the graph database, along with the four-dimensional classification, and are critically synthesized in the Results and Discussion sections. A formal risk-of-bias tool was not applied due to the conceptual and methodological heterogeneity of the included studies; this limitation is acknowledged (Figures 5–10).

Although a formal risk-of-bias or quality assessment tool was not used due to the conceptual heterogeneity of the included studies, several measures were implemented to minimize potential bias. First, the inclusion and exclusion criteria were defined prior to constructing the main corpus, ensuring that the screening process did not adapt to the emerging dataset. Second, the Boolean search strings and domain-specific query structures were pre-specified to avoid selective retrieval. Third, all screening decisions, classifications, and coding assignments were independently reviewed in a cross-checking process by both authors to ensure consistency and reduce subjective bias. Finally, the graph-based Neo4J representation provides a dynamic and transparent structure that enables external readers to inspect, query, and update the dataset, thereby offering a mechanism for continuous correction of potential classification biases. These steps collectively reduce selection, classification, and confirmation biases, even in the absence of a formal risk-of-bias tool.

## 3 Results

### 3.1 Organization

In this review, we searched for research papers across different domains as described in the methodology section, in which all involved the intersections of privacy and AI. Several dimensions of analysis for the papers were considered: the technological domain, the privacy actions described in the paper, the privacy approach, and the AI-privacy relation. As mentioned in the previous section and further explained, the different values of these dimensions are not disjoint sets, i.e., there might be several domains, approaches, etc., in each paper. Nevertheless, to organize the papers for this review, the leading value of each paper in a certain dimension was chosen. The main value was the technological domain, but its only meaning was for sorting. Obviously, textual information can only be displayed according to a single sort of key, however, the graph representation is not subject to this constraint. This choice of organization allows us to identify patterns and trends within specific technologies, making it easier to understand how privacy concerns and solutions are applied in each area. By categorizing papers based on their primary technological domain, we can draw insights into how certain domains, such as healthcare, autonomous systems, or social networks, handle privacy challenges differently. Furthermore, this structure enables a more focused comparison of AI and privacy intersections within each domain, highlighting both the commonalities and unique approaches across different fields. The textual description below (section 3.2) includes a review of each technological domain. A more detailed description is available directly from the graph through a link from each domain node to a PDF file. The different values of the technological domain dimension are: ML, NLP, LLMs, computer vision, speech recognition, IoT, OSN, and DBs.

### 3.2 Review of the papers by the selected dimensions

To complement the four-dimensional classification and to enhance the clarity of the cross-domain findings, we synthesized the main privacy threats and corresponding defense mechanisms identified in the reviewed papers. Table 2 provides a consolidated overview that maps each AI technological domain to the dominant privacy risks reported in the literature, alongside representative mitigation techniques. This summary illustrates how privacy challenges manifest differently across domains, ranging from inference attacks in ML, to re-identification risks in computer vision and sensitive-attribute leakage in speech and NLP systems, while also showing the diversity of defensive strategies, such as differential privacy, federated learning, feature obfuscation, and encryption-based methods. This table supports the interpretive aspects of the review by offering a domain-level comparison and highlighting recurring patterns that emerge across heterogeneous AI applications. The table presents the main components of the classification model and the distribution of papers across its dimensions, supporting the discussion section.

**Table 2:** List of AI domains, associated privacy threats, and representative defense mechanisms.

AI domain	Primary privacy threats	Representative defenses/mitigations
Machine learning (ML)	Model inversion, membership inference, adversarial leakage, and repurposing of training data	Differential privacy (DP), federated learning, homomorphic encryption, secure multiparty computation (SMC), adversarial training
Natural language processing (NLP)	Sensitive text disclosure, metadata leakage, and unintentional extraction of private information from corpora	Data minimization, redaction and automated sensitive-entity detection, DP text perturbation, secure fine-tuning pipelines
Large language models (LLMs)	Prompt-based data extraction, jailbreaks, “model leaks” of training data, hallucinated sensitive personal information	Alignment and RLHF safety filters, prompt-injection defenses, DP-based training, gated access to system prompts, safety guardrails
Computer vision	Facial recognition re-identification, inference of sensitive attributes, surveillance, and tracking	Obfuscation (blur, pixelation), adversarial perturbations, edge-based processing, privacy-preserving feature extraction
Speech recognition	Voiceprint re-identification, inference of emotional/health states, and dataset over-collection	Voice anonymization, local/on-device models, DP feature extraction, consent-based capture policies
IoT	Continuous data harvesting, location tracking, cross-device inference, unauthorized profiling	Access-control architectures, lightweight encryption, FL on constrained devices, blockchain-based auditability
Online social networks (OSN)	Profiling, behavioral prediction, cross-platform identity linkage, and synthetic data attacks	Privacy-aware recommender design, usage of PETs (SMC/DP), user-centric data controls, algorithmic transparency
Databases	Re-identification through linkage attacks, attribute disclosure, and deanonymization	K-anonymity, l-diversity, t-closeness, data perturbation, cryptographic query processing

The mapping presented in Table 2 also demonstrates the dual role of AI across domains, as both a vector for privacy violations and a mechanism for privacy preservation. For instance, while domains like LLMs and IoT introduce substantial risks due to large-scale data aggregation and continuous data capture, they likewise host some of the most advanced privacy-preserving technologies, including federated learning, on-device processing, and cryptographic computation. By summarizing threats and defenses in a single structure, the table reinforces the necessity of domain-specific approaches to privacy protection. It underscores the importance of adaptive regulatory and technical frameworks.

In the rest of this subsection, each domain is described in detail.

#### 3.2.1 Machine learning (ML)

Oseni et al. (2021) reviewed security and privacy challenges in AI, including secure development, adversarial threats, and defense methods. Dilmaghani et al. (2019) surveyed and discussed the impact of big data on privacy and security in the context of ML and AI systems. Ma et al. (2023) surveyed privacy and security issues that can arise in distributed ML systems. Perino et al. (2022) examined vulnerabilities of AI models in telecom, exploring privacy-preserving techniques like Federated Learning (FL), differential privacy, and trusted execution environments. Thuraisingham (2020) discusses the potential of AI to benefit humanity while also acknowledging the challenges of cyber-attacks and privacy violations. Zhang et al. (2022) proposed a taxonomy of explainable AI (XAI) for cybersecurity across domains such as phishing and malware. Khalid et al. (2023) surveyed privacy-preserving machine learning (PPML) in healthcare, detailing attack types and defense techniques. Meurisch et al. (2020) introduced PrivAI, a privacy-by-design platform using divisible AI and confidential data stores. Gupta et al. (2020) investigated AI-enhanced smart contract privacy and identified unresolved challenges in security and scalability. Villegas-Ch and Garcia-Ortiz (2023) proposed a framework to address AI privacy gaps, emphasizing policy and risk mitigation. Gulmezoglu et al. (2019) investigated attacks on mobile devices that exploit the microarchitecture of modern processors and compromise user privacy. Cheng et al. (2020) demonstrated FL in finance and edge computing through real-world deployments. Zhu et al. (2020) analyzed the benefits of differential privacy in ML, Deep Learning (DL), and multi-agent systems. Qiu et al. (2019) surveyed recent progress in adversarial attacks and defense technologies, mainly in DL. Zhou et al. (2022) surveyed current research on adversarial attacks and defenses in DL. Rodriguez-Barroso et al. (2020) introduced Sherpa.ai, combining FL and differential privacy in a unified framework. Jain and Ghanavati (2020) explored privacy risks from large AI training datasets and reviewed emerging solutions. Murdoch (2021) discussed AI in the field of healthcare. Mahmud et al. (2022) addressed autism care using explainable and privacy-preserving AI. Bai et al. (2021) developed a FL framework for COVID-19 diagnosis using chest CT data to preserve privacy without central data sharing. Kaissis et al. (2020) reviewed secure AI techniques in medical imaging, including homomorphic encryption and multi-party computation. Roy (2022) examined data protection methods like anonymization and encryption in healthcare AI under HIPAA and GDPR. Tom et al. (2020) evaluated privacy challenges in AI-driven ophthalmology, covering consent and data protection models. Bak et al. (2022) discussed balancing privacy with access to health data for AI research across the EU. Fritchman et al. (2018) presented a secure multiparty computation (SMC) framework for encrypted AI predictions in clinical settings. Kirienko et al. (2021) compared distributed learning to centralized ML for privacy-sensitive medical applications. Puiu et al. (2021) applied homomorphic encryption to cardiovascular imaging and emphasized explainability in clinical AI. Ishii (2019) compared privacy laws across countries in relation to AI-powered robots and proposed policy suggestions. Willems et al. (2023) found that usefulness of AI services increases adoption despite privacy concerns, supporting the privacy paradox. Zhdanov et al. (2022) proposed integrating fairness, accountability, and transparency (FAT) with privacy in business AI decision-making. Wang et al. (2019) applied differential privacy to aggregate public moral preferences in autonomous vehicle ethics. Tucker et al. (2018) analyzed AI and privacy economics, focusing on data persistence, repurposing, and spillovers. Toch and Birman (2018) introduced a behavioral privacy framework to address AI’s predictive capabilities. Rahman et al. (2020) proposed a privacy-preserving AI framework for edge computing using Fully Homomorphic Encryption (FHE) to protect quality-of-service data. McStay (2020)reviewed emotional AI practices and called for regulation to safeguard dignity and non-identifying data. Roemmich et al. (2023) studied U.S. workers’ reactions to emotion AI and found it raised significant concerns about emotional privacy. Zhang et al. (2021) surveyed AI from the perspective of ethics and privacy. Saura et al. (2022) explored governmental use of AI for behavior analysis and its privacy implications, recommending ethical data practices. Bendechache et al. (2021) described a project designed to engage 500 Dublin teenagers in workshops to explore AI, ethics, and privacy. Chen (2020) explored personal privacy protection techniques from the standpoint of social engineering. Kelley et al. (2023) surveyed global public concerns around AI, such as state surveillance and unauthorized data use. Kronemann et al. (2023) examined how AI personalization and anthropomorphism impact user willingness to disclose data under privacy concerns. Kerry et al. (2020) emphasized the urgency of privacy legislation in response to AI’s transformative data use. Hu et al. (2023) argued for national data infrastructure as a cybersecurity imperative in AI governance. Mazurek and Malagocka (2019) discussed evolving attitudes toward AI, privacy, and regulation, emphasizing ethical alignment. Holzinger et al. (2021) linked AI to the UN Sustainable Development Goals, noting the dual risks and benefits of AI. Ahmadi Mehri and Tutschku (2017) proposed virtual premises for privacy-by-design in cloud-based AI marketplaces to enhance trust. Al-Khassawneh (2023) provides a comprehensive overview of adversarial attacks against AI applications. Amaral et al. (2021) discuss the use of AI to automate the examination of the completeness of privacy policies in compliance with the GDPR. Humerick (2017) focused on the problematic aspects of the implementation of the GDPR together with the development and use of AI. Bae et al. (2018) examined the vulnerability of deep learning models to security and privacy threats, including evasion and poisoning attacks.

#### 3.2.2 Natural language processing (NLP)

Contissa et al. (2018) explored automating legal assessments of privacy policies under the General Data Protection Regulation (GDPR) using ML. Analyzing 14 policies, they found none fully compliant, citing vague language. Though automation showed promise, more training data is needed. Martinelli et al. (2020) proposed a method using AI and NLP to identify sensitive data in unlabeled documents from fields like healthcare and justice. Their semi-automated approach—relying on techniques like word embedding and topic modeling—achieved strong performance on 10,000 documents, with human validation at the end of the loop. Xing et al. (2023) compared AI-related privacy concerns in the U. S. and China using Twitter and Weibo data. Americans were more wary of AI privacy threats, while Chinese users expressed greater optimism—differences attributed to cultural perspectives and views on security and technology. Zarifis et al. (2021) investigated trust in AI-driven health insurance services. Users were less trusting when AI was visible in the interface, suggesting that transparency in AI deployment may reduce perceived trust, even if not significantly. Shahriar et al. (2023) reviewed privacy risks across the AI lifecycle, including ML, expert systems, and NLP. They categorized risks into identification, inaccuracy, non-transparency, and regulatory non-compliance. Their framework promotes a privacy-by-design approach using Privacy-Enhancing Technologies (PETs) and calls for improved transparency, explainability, and adherence to privacy regulations like the GDPR.

#### 3.2.3 Large language models (LLMs)

While Large Language Models (LLMs) are a subset of NLP, their impact warrants separate treatment. Li et al. (2023) examined privacy attacks targeting ChatGPT and Bing, focusing on prompt-based attacks to extract personal data. Their experiments demonstrated successful data leakage through these methods. Gupta et al. (2023) explored the cybersecurity implications of generative AI, especially ChatGPT, highlighting vulnerabilities exploited through jailbreaks, prompt injections, and reverse psychology. They warned of threats including phishing, automated hacking, and malware creation. Proposed solutions include secure code generation, cyber defense automation, attack detection, and ethical guidelines. Mylrea and Robinson (2023) introduced an AI trust framework using an “entropy lens” from information theory to improve transparency in opaque (“black box”) systems. Their model supports trust in human-AI collaboration and was validated through case studies. Wei and Liu (2024) addressed trust in distributed AI, emphasizing robustness, privacy, fairness, and the importance of governance to mitigate distributed learning vulnerabilities. Peres et al. (2023) investigated ChatGPT’s use in mental health, highlighting benefits like generating therapy notes and assessments, but also stressing privacy, accuracy, and ethical risks. They call for strong testing and ethical oversight in sensitive AI applications.

#### 3.2.4 Computer vision

Ferm et al. (2022) examined AI and its impact on consumer privacy, highlighting how technologies like NLP, ML, and DL collect data and raise privacy concerns. Through case studies such as Clearview AI and Hello Barbie, they stressed the need for ethical, transparent data handling. Harichandana et al. (2022) introduced a lightweight AI framework to detect sensitive content in images of people with disabilities, using object and eye-gaze detection to prompt consent before sharing, emphasizing ethical photography practices. Liu et al. (2019) proposed a privacy-preserving method for images, combining adversarial perturbations (against AI) with visual obfuscation (against humans). Their approach protects against both human and AI attackers analyzing multimedia.

#### 3.2.5 Speech recognition

Curzon et al. (2021) classified AI privacy risks across five domains: computer vision, speech recognition, NLP, knowledge representation, and automated reasoning. They proposed mitigation techniques for each to reduce vulnerabilities. Li and Zhang (2017) explored privacy, security, and ethics in AI applications such as speech input and intelligent shopping systems, analyzing the distinct risks in each category. Liu et al. (2021) reviewed ML and privacy in speech recognition, outlining how ML can be both a privacy threat and a protective tool. They proposed a three-part framework: ML as a privacy target, as a defense, and as an attacker, and highlighted open research gaps. Gandeeban et al. (2025) introduced SER-EQCNN-ESC, a new architecture for Speech Emotion Recognition (SER). It integrates Bellman Filtering for noise reduction, Holistic Dynamic Frequency Transformer for feature extraction, and a Single Candidate Optimizer. Final classification uses an Equivariant Quantum Convolutional Neural Network (EQCNN), optimized via the Educational Competition Optimization algorithm, enhancing SER accuracy and robustness.

#### 3.2.6 Internet of things (IoT)

Giordano et al. (2022) reviewed how AI supports privacy in Internet of Things (IoT) systems, organizing literature across dimensions like algorithms, datasets, and evaluation strategies. Elhoseny et al. (2021) proposed a blockchain-based, AI-enabled IoT system for private healthcare data transfer, demonstrating improved security and performance. Xiao et al. (2018) surveyed Machine Learning (ML) approaches for IoT security, including authentication and malware detection, and recommended transfer learning to address IoT devices’ limited context awareness. Liu et al. (2022) examined privacy concerns around smart speakers in China using a socio-technical framework, highlighting the role of regulation, especially the Personal Information Protection Law (PIPL). Jin et al. (2018) analyzed how AI and big data impact consumer privacy in IoT transactions, emphasizing the dual role of data in improving services and increasing risks. Manheim and Kaplan (2019) explored how AI challenges privacy, democracy, and transparency, and suggested regulatory responses. Tschider (2018) criticized existing legal frameworks for consumer IoT, calling for better privacy and discrimination protections. Van Van Hoven Genderen (2017) questioned the adequacy of GDPR in addressing AI’s growing role in personal data use. Xiong et al. (2019) introduced an AI-based game-theoretic model to protect privacy in mobile edge crowdsensing (MECS), showing its effectiveness in simulations. Keshta (2022) addressed privacy challenges in AI-powered IoT (AIIoT) for healthcare, stressing the need for architectural standards. Sun et al. (2020) examined the role of ML in balancing privacy and performance in 6G networks. Gray and Mehrnezhad (2025) propose PhotonKey, a lightweight key pairing system for constrained IoT devices that uses ambient light sensor data as shared input for secure symmetric key generation. Sedenberg and Chuang (2017) warned of emotional AI’s ability to manipulate without consent and called for regulatory safeguards. Schiliro et al. (2020) proposed an AI approach using DL to preserve privacy in brainwave (EEG) data. Sachdev (2020) explored the security-privacy trade-off in Edge AI for digital marketing. Sugianto et al. (2024) designed a privacy-preserving public surveillance system using federated learning within their Responsible AI Implementation Framework (RAIFF). Gao et al. (2023) proposed a game-theoretic model to balance PPDM and cloud-based IoT data usage. Awad et al. (2024) reviewed AI-enhanced biometric authentication in IoT, focusing on facial and fingerprint recognition. Yepuganti et al. (2021) developed an IoT plant-monitoring system that aids mental health via interactive gardening using cloud and voice assistant technologies. Rana et al. (2025) provide a comprehensive review of machine learning applications in the Internet of Nano Things, highlighting current challenges and outlining future research directions.

#### 3.2.7 Online social networks (OSN)

Sattikar and Kulkarni (2012) highlight how AI techniques—such as neural networks, genetic algorithms, and fuzzy logic—can address privacy and security issues in Online Social Networks (OSN) by reducing subjectivity in assessments.

Hirschprung and Alkoby (2022) introduced the Online Information-Sharing Assistance (OISA) framework, using game theory and AI agents to help users weigh privacy risks when sharing information online. Simulations showed OISA agents outperformed humans in maximizing utility. Wang et al. (2021) modeled privacy dynamics in AI-driven e-commerce using evolutionary game theory, recommending strategies for balancing personalization with privacy. Majeed and Hwang (2023) warned of AI-generated synthetic data undermining anonymization, calling for stronger privacy protections. Wang et al. (2022) surveyed the metaverse’s security and privacy challenges, stressing the need for scalable, interoperable solutions. Subramanian (2017) examined legal and ethical issues of social robots, emphasizing the risks of emotional manipulation and privacy breaches. Cheng and Jiang (2020) found that while chatbots enhance satisfaction through utility and entertainment, privacy concerns reduce trust and loyalty.

#### 3.2.8 Databases (DBs)

Devi (2023) outlines core PPDM methods such as anonymization and cryptography, noting their broad applicability beyond healthcare. Hewage et al. (2023) emphasize the trade-off between privacy and accuracy and extend the discussion to Privacy-Preserving Data Stream Mining (PPDSM), which adds real-time and resource challenges. Hirschprung (2023) categorizes PPDM techniques into anonymization, randomization, cryptography, and result privatization, each addressing privacy through different mechanisms.

### 3.3 Neo4J graph representation

To complement the narrative results above, we provide a graph-based visualization of the classified evidence, which enables a structural understanding of cross-domain relationships and supports evidence mapping.

As described in the Methodology section, Neo4J is a Graph Database Management System (GDBMS), designed to efficiently store, query, and analyze relationships between data. Unlike traditional Relational Databases (RDB), which are based on tables, Neo4j represents data as Graphs. Though some examples are described below, real visualization should be done digitally. As mentioned above, all the data and models are available on GitHub (Voloch, 2024).

To improve clarity, it is important to note that the Neo4J visualizations serve primarily as illustrative snapshots of the underlying graph structure. Their full value is realized in the interactive environment, where users can zoom, filter nodes, explore specific subgraphs, and examine relationships dynamically. The figures included in the manuscript are simplified static representations intended only to demonstrate the structure and logic of the evidence-mapping approach.

Figure 5 presents all the reviewed papers with all of their links to their specific dimension values for all of the different dimensions and values in the Neo4J DB. As can be seen, many of the papers have more than one value in a specific dimension and a link to more than one dimension. This inherent trait of research review data demonstrates the advantage of graph representation. Naturally, when the full graph is presented on paper, it is overloaded, but when viewed digitally, it can be zoomed, cropped, and most importantly queried. Because the Neo4J visualizations represent a high-dimensional graph with many interconnected nodes, the full graph can appear dense when shown as a static figure. The intention is for readers to explore the graph interactively in the Neo4J environment, where zooming, filtering, and querying functions allow clear inspection of specific components. The static figures included in the manuscript therefore, serve as simplified illustrative snapshots rather than full-resolution analytical views.

Figure 6 illustrates all of the reviewed papers with all of their links to their domains’ values. As seen, a considerable number of papers are in the domains of *ML*, *OSN,* and *IoT*. It is also apparent that papers that are classified with the DB value do not share other values.

Figure 7 provides a visualization of all the papers reviewed with all of their connections to their specific action values. As seen, in many of the papers, there are several actions presented, as the graph connectivity is high for most of the action nodes.

Figure 8 presents a visualization of all the papers reviewed with all of their connections to their specific approach values. As can be seen, the most connected approach nodes are the *PbD* and *Advisory* ones. The graph demonstrates very high connectivity in these nodes.

Figure 9 exhibits a visualization of all the papers reviewed with all of their connections to their specific direction values. As seen, the most connected approach nodes are those related to AI as a threat to privacy and the application of *privacy to AI*. The graph depicts very high connectivity in reference to these nodes.

The use of Neo4J to implement a graph enables complex queries that are based on links. The query can be implemented manually by observing the graph (since links pop out from a graph, unlike RDB), or by entering a code.

For example, a query code to find papers that are in the *ML* domain, that include the Action of *Defense*, follow the PbD approach, and their AI-privacy direction is *Applying privacy to AI*, will be:

MATCH (p: Paper)-[: BELONGS_TO]- > (d: Domain {description: “ML”}).
MATCH (p: Paper)-[: BELONGS_TO]- > (a: Action {description: “Defense”}).
MATCH (p: Paper)-[: BELONGS_TO]- > (ap: Approach {description: “Privacy by Design (PbD)”}).
MATCH (p: Paper)-[: BELONGS_TO]- > (r: Relation {description: “Applying privacy to AI”}).
RETURN p, d, a, ap, r;

The result of this query is depicted in Figure 10.

**Figure 11:**
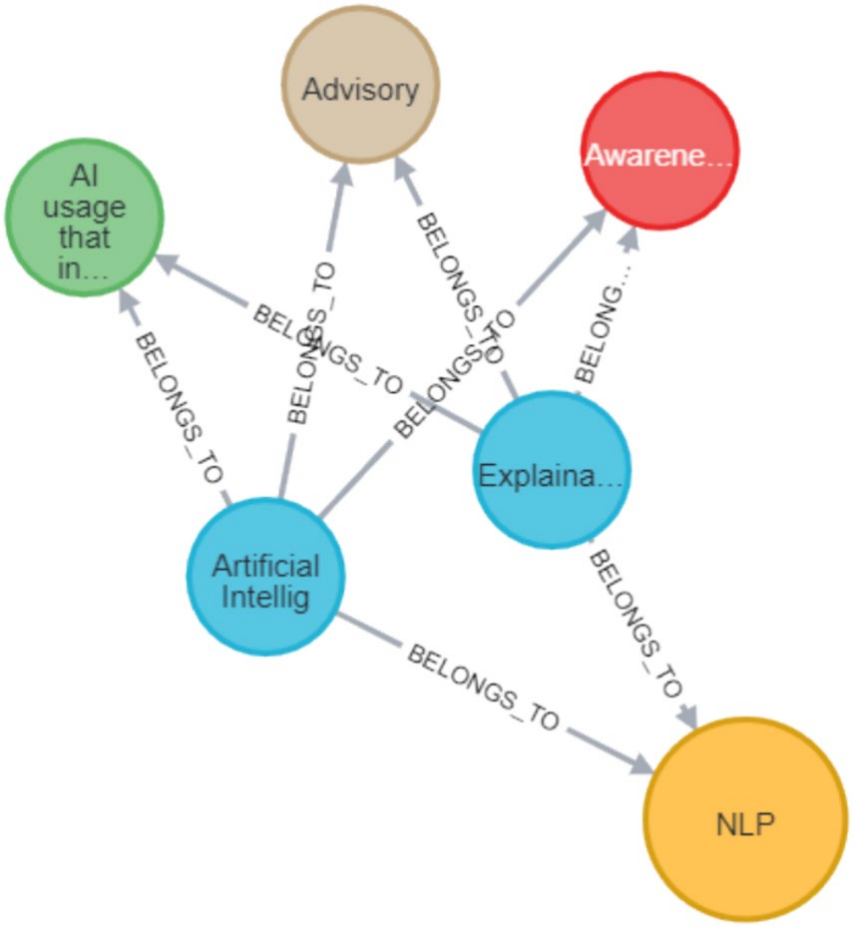
A graphic representation produced from querying the GDB for: all the papers that are in the *NLP* domain, that include the action of a*wareness*, follow the a*dvisory* approach of, and whose AI-privacy direction is *AI usage that includes privacy*.

Another example, a query code to find papers that are in the *NLP* domain, includes the Action of *Awareness*, follow the approach of *Advisory*, and their AI-privacy direction is *AI usage that includes privacy*, is:

MATCH (p: Paper)-[: BELONGS_TO]- > (d: Domain {description: “NLP”}).
MATCH (p: Paper)-[: BELONGS_TO]- > (a: Action {description: “Awareness”}).
MATCH (p: Paper)-[: BELONGS_TO]- > (ap: Approach {description: “Advisory”}).
MATCH (p: Paper)-[: BELONGS_TO]- > (r: Relation {description: “AI usage that includes privacy”}).
RETURN p, d, a, ap, r;

The result of this query is depicted in Figure 11. The visualization of the query shows that there are only two papers that comply with these conditions.

**Figure 12:**
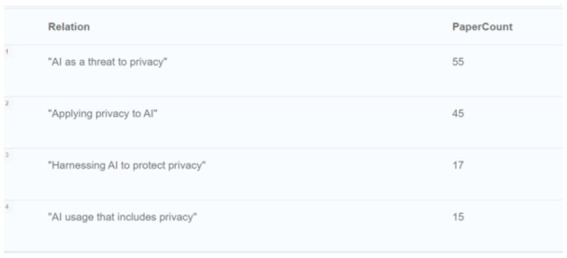
A list representation produced from querying the GDB for: All the papers that are in each relation direction for the papers that are in the *ML* domain.

Another example, a query code for counting how many papers are in each Relation Direction for the papers that are in the *ML* domain and presenting them as a list, is:

MATCH (p: Paper)-[: BELONGS_TO]- > (: Domain {description: “ML”}).
MATCH (p)-[: BELONGS_TO]- > (r: Relation).
RETURN r.description AS Relation, COUNT(p) AS PaperCount.
ORDER BY PaperCount DESC;

The result of this query as a list in Neo4J is depicted in Figure 12.

### 3.4 Role and value of the graph-based representation

The Neo4J graph representation serves as an evidence-mapping tool that complements the qualitative synthesis by revealing structural patterns that are not easily detectable through narrative review alone. Unlike traditional tables or taxonomies, the graph exposes the network relationships between Domains, Actions, Approaches, and Directions. This allows readers to observe clusters of research activity, identify sparsely explored intersections, and trace how specific privacy mechanisms (e.g., differential privacy, homomorphic encryption) appear across multiple AI subfields.

A key advantage of the graph is that it provides a queryable, extensible, and reproducible structure. Researchers can explore the graph interactively by filtering nodes, isolating domain clusters, or examining paths between concepts (e.g., linking “LLMs” to “Inference Attacks” to “Mitigation Techniques”). This interactive format enables future work such as locating underrepresented privacy risks, validating emerging research trends, or adding new papers to extend the map beyond the scope of the present review.

However, representing heterogeneous literature in a graph format also has limitations. Some conceptual nuances may be simplified when encoded as discrete nodes, and the density of interconnected nodes can appear visually complex in static figures. For this reason, static illustrations in the manuscript serve only as high-level demonstrations, while the intended use of the graph is through the interactive Neo4J environment, where zooming, filtering, and node-level inspection mitigate visual complexity. The graph should therefore be interpreted as a structural overview rather than a replacement for detailed methodological comparison.

## 4 Discussion

The integration of AI into various domains of our lives introduces both benefits and costs, particularly concerning privacy. Privacy violation in the digital age may be divided into three stages: (a) When Information Systems began to appear and large databases that contain sensitive personal data could leak into the wrong hands (Shu et al., 2015); (b) When the Internet appeared more and more sensitive personal data began to be published by the data owner himself (Lippert and Swiercz, 2007); and (c) When the AI began to appear, and sophisticated processes could access sensitive information that apparently is not currently available. Therefore, in this review, we aimed to introduce the current research on this significant issue (Liu et al., 2020).

In this review, eight technological domains were analyzed. The results show apparent, systematic differences across the various domains, which are reflected in the nature of privacy risks and the maturity of defenses. Machine Learning (ML) and Large Language Models (LLMs) exhibit the most advanced and well-documented attack surfaces, particularly model inversion, membership inference, and prompt-based data extraction. In contrast, IoT and Online Social Networks (OSNs) concentrate on continuous behavioral surveillance and cross-device/cross-platform inference. Unlike ML and LLMs, where differential privacy, federated learning, and cryptographic computation are widely studied, IoT and speech systems still rely heavily on architectural and policy-based mitigation due to constraints on computational resources or real-time processing. Computer vision presents a unique duality: the same models enable both highly intrusive identification and highly effective privacy-preserving transformations. These cross-domain contrasts indicate that privacy in AI is not monolithic but shaped by data modality, system architecture, and operational constraints. For this reason, the review is multi-dimensional.

### 4.1 Synthesis across domains

Taken together, the results of this scoping review reveal several cross-cutting patterns across the literature. First, privacy risks tend to cluster in domains where data granularity is high, such as NLP, computer vision, and OSN, where inference attacks and unintended disclosures are especially prominent. Second, the review identifies a consistent tension between technical mitigation strategies (e.g., differential privacy, federated learning, homomorphic encryption) and their uneven adoption in applied research. Third, conceptual and regulatory discussions increasingly emphasize socio-technical themes, highlighting that effective privacy protection requires not only technical safeguards but also accountability, transparency, and governance structures. Finally, the graph-based evidence map illustrates that privacy challenges are not isolated within a single domain, but form interconnected patterns that span modalities and application areas, underscoring the need for integrated and cross-domain privacy approaches in future AI research.

Based on the findings in this review, AI presents a dual role in privacy, both as a tool for enhancing privacy and as a technology that introduces new privacy risks. Techniques such as federated learning, differential privacy, and homomorphic encryption demonstrate how AI can be harnessed to preserve privacy (Padmanaban, 2024). Nonetheless, the ability of AI to infer, predict, and process large amounts of personal data, often without explicit user consent, poses significant concerns (Tene and Polonetsky, 2012).

The number of papers on the topic of AI and privacy is vast, and consequently, we categorized the papers into four dimensions: The technological *domains* of a research paper, which define the specific field of technology it addresses, including relevant methods, tools, applications, and innovations central to its focus. The domains identified include LLMs, ML, NLP, computer vision, speech recognition, IoT, OSNs, and DBs; Privacy *actions*, which in the paper refer to the measures, techniques, or strategies proposed or analyzed to protect data privacy, mitigate risks, or address privacy-related challenges in cybersecurity, covering attacks, defense, awareness, vulnerabilities, threats, and regulations.

The AI-privacy relation direction indicates how the study explores or addresses the interaction between AI and privacy, considering implications, challenges, and opportunities, with identified directions including harnessing AI to protect privacy, AI as a threat to privacy, AI usage that incorporates privacy, and applying privacy principles to AI. As observed in this review, there are different approaches to privacy in AI, including: PPDM, privacy by design, privacy shell, advisory, and hybrid methodologies that integrate multiple protective measures. Some studies highlight AI’s potential to secure privacy, particularly in applications like healthcare and social networks, where sensitive data is regularly processed (Valente, 2010). At the same time, adversarial attacks, data breaches, and privacy vulnerabilities continue to challenge existing privacy-preserving frameworks (Xu et al., 2021) AI models trained on large datasets remain susceptible to extracting unauthorized information, raising ethical and regulatory concerns.

Legal frameworks such as the GDPR provide mechanisms to mitigate privacy risks, yet the rapid evolution of AI often outpaces regulatory updates, especially considering the conservative and slow-paced legal process. Ensuring compliance while maintaining AI efficiency remains a critical issue (Balakrishnan, 2024). Transparency and explainability in AI models are also vital, as they influence trust and accountability in privacy-sensitive applications. Addressing bias in privacy-preserving AI models is another challenge that requires further attention to ensure that privacy protection does not inadvertently reinforce discrimination or unequal access.

To further connect the technical findings of this review with established normative and regulatory frameworks, we highlight how the observed privacy threats and defenses align with key global principles such as the GDPR and the OECD AI Principles. Many of the technical risks identified, such as model inversion, attribute inference, unauthorized profiling, and cross-device tracking, directly correspond to GDPR provisions on data minimization (Art. 5), purpose limitation (Art. 6), data protection by design and by default (Art. 25), and restrictions on automated decision-making (Art. 22). Likewise, the defense mechanisms highlighted in this review including federated learning, differential privacy, homomorphic encryption, and privacy-aware model training, operationalize GDPR’s requirements for privacy-by-design and accountability. In parallel, the OECD AI Principles emphasize transparency, robustness, and human-centric design. At the same time, our synthesis shows that privacy-preserving techniques, such as explainable AI, adversarial robustness, on-device processing, and secure computation, directly support these normative objectives. By linking the technical landscape of AI privacy with these regulatory frameworks, the review demonstrates how emerging privacy-enhancing technologies can serve as practical tools for achieving compliant, trustworthy, and responsible AI ecosystems. This interdisciplinary mapping underscores the need for ongoing collaboration between technical researchers, policymakers, and organizations implementing AI systems.

A comparative view across the reviewed studies reveals notable differences in the strengths and limitations of primary privacy-preserving techniques. Federated Learning (FL) reduces the need to centralize data and is effective for distributed or edge-based environments; however, several papers have reported persistent risks of gradient leakage and model inversion attacks. Differential Privacy (DP) provides formal privacy guarantees and is particularly effective for statistical and text-based tasks; however, its performance degradation on complex deep learning models remains a recurring challenge. Homomorphic Encryption (HE) enables computation over encrypted data and aligns strongly with high-assurance privacy requirements; however, its computational overhead and lack of scalability for large models are frequently cited as limitations. These findings suggest that no single technique is universally optimal; rather, the literature increasingly points toward hybrid approaches that combine FL, DP, HE, or secure multiparty computation, each compensating for the weaknesses of the others.

The synthesis also reveals structural gaps in the literature. First, most domains treat privacy attacks and defenses independently rather than as interacting systems. For instance, ML research develops mathematical defenses (DP, HE), while IoT research emphasizes regulatory constraints and device-level access control, with limited cross-pollination. Second, few papers integrate technical defenses with human-centric concerns such as trust, behavioral biases, and user-facing transparency mechanisms, despite their prominence in OSN and emotion-AI studies. Third, LLM research is disproportionately focused on jailbreaks and data extraction, while offering relatively fewer evaluations of long-term privacy leakage through fine-tuning, model updates, or synthetic data pipelines. Finally, few studies examine multi-domain attack chains. However, the evidences suggest that these domains increasingly interoperate. These gaps indicate fragmentation and highlight the need for unified frameworks that bridge technical, behavioral, and regulatory perspectives across domains.

### 4.2 Limitations of the review

The literature also shows several gaps and contradictions. While many studies emphasize the effectiveness of DP for mitigating inference attacks, others report that DP may be insufficient against sophisticated attribute reconstruction techniques in high-dimensional models. Similarly, although FL is often portrayed as privacy-preserving, empirical studies highlight that decentralized training can amplify certain vulnerabilities, contradicting assumptions that “no raw data leaves the device” is sufficient protection. A further gap is the limited evaluation of real-world deployment constraints, most privacy-preserving techniques are tested under idealized laboratory settings rather than industry-scale workloads. Finally, inconsistencies in threat modeling across papers make it difficult to compare results, underscoring the need for standardized benchmarks and reporting practices.

These comparative and cross-domain insights have direct implications for policy and system design. From a regulatory perspective, hybrid PET architectures better align with the GDPR’s requirements for data minimization, privacy by design, and accountability. For system designers, the contradictions in reported performance highlight the importance of context-dependent PET selection, where threat models, resource constraints, and domain-specific risks guide the choice of FL, DP, HE, or alternatives. Ethically, the persistent vulnerabilities in LLMs, OSNs, and IoT systems underscore the need for continuous monitoring, transparent communication of privacy risks, and auditability mechanisms. Taken together, the findings argue for an integrated socio-technical approach where technical safeguards are reinforced by regulatory frameworks and organizational governance.

From a practical standpoint, the findings carry implications for system designers, regulators, and applied researchers. For developers of ML and LLM systems, the evidence suggests that privacy-preserving techniques (DP, FL) are mature but unevenly adopted; integrating them as default components, rather than optional add-ons, remains an urgent priority. For IoT and OSN ecosystems, architectural decisions (e.g., on-device processing, data minimization, and decentralization) offer more realistic privacy gains than post-hoc analytics, given the real-time and continuous nature of data collection. Regulators should note that AI-specific privacy harms, such as inference attacks and cross-modal attribute extraction, are largely unaddressed by current legal frameworks, including the GDPR and HIPAA, which focus primarily on data collection and storage rather than algorithmic inference. Finally, researchers should prioritize evaluating hybrid attack-defense dynamics, particularly how new generative models increase systemic risk when interacting with older systems in healthcare, finance, or IoT workflows.

The organization and classification of research in this review illustrate how different AI domains handle privacy issues uniquely. While federated learning and differential privacy serve as viable solutions in some applications, they introduce computational overhead and utility limitations (Cheng et al., 2020). Similarly, privacy concerns in LLMs, IoT, and computer vision require domain-specific adaptations of existing privacy-preserving techniques (Hewage et al., 2023). The use of graph database technology (with the Neo4J DB engine) in this review enhances the understanding of privacy relationships within AI by enabling a clearer depiction of how privacy actions, technological domains, and AI applications intersect. Moreover, it enables querying the research according to any filter or sort order as selected by the reader (when accessing the DB itself, which is publicly available), a feature that is not applicable in a review that is provided only textually. The reader can not only apply smart search to the research, but can also update it, e.g., when a new paper appears.

The novelty of this research is that a different perspective of review is offered, with a deeper analysis of the papers’ data, in the domains mentioned above. The analysis was performed after creating the raw DB, and the categorization was then assigned. This analysis is extended to the internal links in the data, a process that contributed to a deeper understanding of the problems and the solutions that are described in the papers reviewed.

Another important consideration is the role of public perception in shaping privacy policies for AI. Users’ trust in AI-driven technologies is crucial for their widespread adoption, and any perceived privacy violations could lead to regulatory pushbacks and reduced engagement.

Despite its contributions, this research has certain limitations. First, while the review covers a broad range of AI applications and privacy concerns, it is inherently constrained by the available literature. Some emerging AI privacy challenges, particularly those associated with novel technologies such as generative AI and quantum computing, may not yet have been extensively studied, limiting the scope of the analysis. Furthermore, the classification and categorization of the reviewed papers, while structured, may be subjective to some extent, as the definitions and interpretations of privacy concerns vary across disciplines.

Another limitation lies in the reliance on publicly available research and databases. Some of the most advanced AI privacy solutions may exist within proprietary systems or unpublished industry research, which are not accessible for review. Additionally, privacy-preserving techniques often involve trade-offs in performance, security, and usability, and this review does not provide empirical validation of these trade-offs. Future research should include experimental validation of privacy-preserving AI techniques to comprehensively assess their real-world effectiveness and limitations.

In addition to the constraints already acknowledged, it is essential to note that the review may still be affected by forms of bias inherent to literature-based analyses. Database bias may occur because of different academic repositories (e.g., IEEE Xplore, ACM, Springer, Scopus, PubMed, Google Scholar) that index distinct subsets of the AI and privacy research landscape, potentially overrepresenting certain domains while underrepresenting emerging or interdisciplinary work. Likewise, publication bias may influence the visibility of studies: research reporting positive results, successful defenses, or novel threats is more likely to be published than studies with null findings or unsuccessful attempts. This imbalance can skew the perceived prevalence of particular privacy risks or mitigation strategies across domains. While the graph-based analysis mitigates some of these effects by revealing structural patterns across the included works, future studies should incorporate broader grey-literature searches and alternative indexing sources to reduce these potential biases further.

### 4.3 Future research directions

Future research that will further extend this review and research may focus on refining privacy-preserving techniques that balance computational efficiency, fairness, and robustness. Collaboration between AI developers, policymakers, and ethicists is essential to ensure privacy remains a core design principle rather than an afterthought. AI-driven privacy protections should not be viewed as a limitation but as an enabler of ethical and responsible AI adoption. The insights from this review contribute to an ongoing discourse on aligning AI advancements with privacy preservation, ensuring that the benefits of AI are realized without compromising individual rights.

Future research can also examine how transparent communication about AI privacy measures can influence user trust and acceptance. Exploring user-centered design approaches that empower individuals with greater control over their data could serve as a valuable strategy in mitigating AI-related privacy concerns while maintaining AI’s practical benefits. In this regard, Explainable AI (XAI) should be given significant attention (Ezzeddine, 2024).

Moreover, as AI continues to develop, the interaction between privacy concerns and AI applications will likely evolve in accordance, necessitating ongoing research. The increasing complexity of AI models, such as large-scale transformers and multimodal AI, will present new privacy risks that are not yet fully understood. As AI becomes more autonomous, questions of data ownership and control will need to be re-evaluated, particularly in cases where AI-generated insights influence real-world decision-making. Future research should explore how AI’s predictive capabilities affect privacy expectations and whether current legal and ethical frameworks are sufficient to regulate these changes.

The novel method presented here of analyzing and presenting the reviewed papers by means of a Graph DB, can be further adopted and used in other reviews, as the principles of categorizations and links are common.

AI is an emerging technology that is rapidly growing and penetrating our lives. The same applies to privacy concerns, which are under the spotlight of public attention. Both AI and privacy are not going anywhere, as well as the tight bond between them, for better or worse. The topic of this review is expected to grow and become increasingly central.

Taken together, the evidence demonstrates that AI is simultaneously a catalyst for new forms of privacy harm and a source of advanced privacy-preserving technology. However, these capabilities develop asymmetrically across domains, resulting in an uneven landscape where some areas, such as LLMs and IoT, are rapidly evolving in ways that outpace existing theoretical frameworks and regulatory protections. A coherent, cross-domain privacy strategy for AI, therefore, requires integrating technical defenses, behavioral insights, and governance mechanisms rather than treating them as isolated solutions.

## Data Availability

The datasets presented in this study can be found in online repositories. The names of the repository/repositories and accession number(s) can be found in the article/supplementary material.
